# Morphological and Molecular Evidence for Infrageneric Relationships in *Blastus* (Melastomataceae)

**DOI:** 10.3390/plants15162526

**Published:** 2026-08-20

**Authors:** Xiaopei Wu, Li Wang, Sijin Zeng, Yan Deng, Zuxing Wei, Jingliao Chen, Zhong-jian Liu, Zhenying Wen, Donghui Peng

**Affiliations:** 1 The Cross-Strait Scientific and Technological Innovation Hub of Flower Industry, College of Landscape Architecture and Art, Fujian Agriculture and Forestry University, Fuzhou 350002, China; xiaopeiwu@fafu.edu.cn (X.W.); 52519052005@fafu.edu.cn (L.W.); yandeng@fafu.edu.cn (Y.D.); fjwzx02@163.com (Z.W.); fjchenjl@fafu.edu.cn (J.C.); 2 Guangdong Key Laboratory for Innovative Development and Utilization of Forest Plant Germplasm, College of Forestry and Landscape Architecture, South China Agricultural University, Guangzhou 510642, China; kaileqixia@gmail.com; 3 Institute of Leisure Agriculture, Jiangsu Academy of Agricultural Sciences, Nanjing 210014, China

**Keywords:** *Blastus* Lour., genome-wide SNPs, phylogenies, morphology, infrageneric relationships, molecular phylogenetics

## Abstract

*Blastus* Lour. is a taxonomically complex genus of Sonerileae (Melastomataceae), with unresolved infrageneric relationships and controversial boundaries among several species and varieties. In this study, we integrated morphological evidence with a broadly sampled internal transcribed spacer (ITS) dataset, an expanded nuclear ribosomal DNA (nrDNA) dataset, plastome data, and genomic single-nucleotide polymorphism (SNP) data to evaluate the phylogenetic relationships and classification within *Blastus*. The broadly sampled ITS and expanded nrDNA analyses supported the monophyly of the sampled *Blastus* accessions but provided limited resolution of relationships among closely related taxa. The plastome analysis did not recover the sampled *Blastus* accessions as monophyletic and revealed marked cytonuclear phylogenetic incongruence. The genomic SNP analysis strongly supported the monophyly of the sampled *Blastus* accessions and recovered the sampled members of the terminal inflorescence group as a monophyletic clade. However, the sampled members of the traditional axillary inflorescence group were not monophyletic because *Blastus borneensis* was recovered as sister to the terminal inflorescence clade. Within the terminal inflorescence clade, the sampled members of the *B. pauciflorus* and *B. cavaleriei* groups formed two strongly supported sister lineages. Morphological comparisons showed that inflorescence position and architecture, the distribution of yellow glands on the abaxial leaf surfaces, and calyx lobe morphology generally corresponded to the principal lineages recovered in the SNP tree, whereas indumentum characters varied considerably among individuals and populations. We therefore recommend retaining the axillary and terminal inflorescence groups as descriptive morphological categories and treating the *B. pauciflorus* and *B. cavaleriei* groups as two informal working subdivisions within the terminal inflorescence group. The broad circumscription of *B. pauciflorus* adopted in the *Flora of China* requires reassessment, whereas the boundaries of *B. longiflorus* var. *apricus* and *B. dunnianus* require broader population sampling before formal taxonomic changes are proposed.

## 1. Introduction

Traditional plant classification has relied largely on morphological characters. However, morphological similarities may result from convergent or parallel evolution, and the relative importance assigned to particular characters may differ among taxonomists. These factors can lead to inconsistent taxonomic treatments and reduce the stability of classification systems [1]. With the rapid development of high-throughput sequencing technologies, molecular systematics has become an increasingly important approach for reconstructing plant phylogenetic relationships and resolving taxonomically complex groups [2,3,4].

Molecular phylogenetic inference relies on patterns of DNA sequence variation among taxa. Nuclear ribosomal DNA (nrDNA), with conserved ribosomal genes and intervening spacer regions, has been widely used in phylogenetic studies and may provide valuable information for resolving relationships among closely related taxa [5]. The internal transcribed spacer (ITS) region is a component of the nrDNA repeat and generally evolves more rapidly than the ribosomal genes, which often makes it informative for resolving relationships among species and within genera [6,7]. Plastomes are relatively conserved in structure, usually straightforward to align, and widely used in plant phylogenetic studies [8,9,10]. Nevertheless, evidence from a single locus or genomic compartment may not fully reflect the evolutionary history of taxonomically complex groups. Genomic single-nucleotide polymorphism (SNP) data capture variations across many regions of the nuclear genome and can therefore provide substantial information for resolving relationships among closely related species and major lineages within genera [11,12]. Integrating nrDNA, plastome, and genomic SNP data can strengthen phylogenetic inference and provide a more complete view of evolutionary relationships. Moreover, because plastomes are generally uniparentally inherited and largely non-recombining, whereas nuclear genomes reflect a predominantly biparental and recombining evolutionary history, comparisons between nuclear and plastid phylogenies can reveal cytonuclear discordance and help generate hypotheses concerning processes such as hybridization, introgression, incomplete lineage sorting, and plastid capture [13,14,15].

*Blastus* Lour. is a genus of Melastomataceae, subfamily Melastomatoideae, tribe Sonerileae. The genus comprises approximately 18 species distributed mainly in tropical and subtropical Asia, from eastern India through Southeast Asia to southern China and the Ryukyu Islands of Japan. China is considered its principal center of diversity [16,17,18,19]. The taxonomic history of the genus is complex, and its tribal placement has varied among classification systems. Vliet placed *Blastus* in subtribe Sonerilinae based on wood anatomical characters [20], whereas Chen assigned it to Oxysporeae [16]. Renner subsequently included Oxysporeae within Sonerileae, and *Blastus* has since generally been treated within this tribe [21]. Previous phylogenetic studies of Melastomataceae and Sonerileae have included *Blastus*, but most sampled only one or a few representative species and focused primarily on the phylogenetic placement of the genus [22]. Consequently, the monophyly of *Blastus* and its relationships with allied genera have not been adequately tested with broad sampling across the genus.

Regarding classification within the genus, Diels recognized two broad morphological assemblages in *Blastus*, distinguished mainly by inflorescence position and petal color [23]. One assemblage comprised taxa with predominantly axillary inflorescences and white petals, including *Blastus cochinchinensis* Lour., *Blastus setulosus* Diels, and *Blastus tenuifolius* Diels. The other comprised taxa with terminal inflorescences and red or purple petals, including *Blastus dunnianus* H.Lév., *Blastus multiflorus* Guillaumin, and *Blastus pauciflorus* (Benth.) Guillaumin [23]. Subsequent taxonomic studies adopted markedly different circumscriptions for several species and infraspecific taxa. Hansen treated *Blastus apricus* (Hand.-Mazz.) H.L.Li, *B. dunnianus* H.Lév., *B. lii* Nayar, and *Blastus spathulicalyx* Hand.-Mazz. as synonyms of *Blastus cavaleriei* H.Lév. & Vaniot and treated *Blastus ernae* Hand.-Mazz. and *Blastus longiflorus* Hand.-Mazz. as synonyms of *B. pauciflorus* [19,24]. The circumscription of *B. pauciflorus* was further broadened in the *Flora of China*, in which *B. longiflorus*, *B. cavaleriei*, *B. dunnianus*, *B. ernae*, *Blastus squamosus*, and several related varieties were all treated as its synonyms [16,24]. In this treatment, the description of *B. pauciflorus* in the *Flora of China* includes terminal paniculate cymes, yellow glands on the abaxial leaf surface, dense pubescence and sparse glands on the inflorescences, hypanthia densely covered with stalked glands, and petals ranging from pink to purplish red. The calyx lobes range from shortly triangular to spathulate and from 0.5 to 3 mm in length, whereas the anther bases range from bituberculate to sagittate. Together, these character ranges indicate that a broad morphological circumscription was adopted for the species. Leaf size, indumentum and gland development, and calyx-lobe shape and length, which had previously been used to distinguish the relevant taxa, were therefore interpreted as intraspecific variation within *B. pauciflorus* sensu lato. However, whether these morphological differences represent intraspecific variation or correspond to distinct evolutionary lineages has not yet been adequately tested using molecular evidence based on broad taxon sampling.

Given these taxonomic uncertainties, the present study combined field observations and comparisons of herbarium specimens with analyses of a broadly sampled ITS dataset, an expanded nrDNA dataset, plastome data, and genomic SNP data to evaluate the phylogenetic relationships and morphological groupings of *Blastus*. Before conducting the molecular analyses, we formulated a morphological hypothesis based on field and herbarium observations. Taxa with terminal inflorescences were provisionally divided into two informal groups, the *B. pauciflorus* group and the *B. cavaleriei* group, according to the distribution of yellow glands on the abaxial leaf surface and the shape and degree of reflexion of the calyx lobes. These groups were used solely as working units for testing the correspondence between morphological differentiation and molecular lineages and do not represent formally established infrageneric taxa. Specifically, this study addressed three questions. First, does expanded sampling within *Blastus*, together with the inclusion of allied genera, support the monophyly of the sampled *Blastus* accessions and clarify their relationships with related genera? Second, are the principal relationships inferred from ITS, nrDNA, plastome, and genomic SNP data congruent, and where do the nuclear and plastid phylogenies conflict? Third, do the traditional morphological assemblages based on inflorescence position and the two informal groups proposed within the terminal inflorescence group correspond to the principal nuclear genomic lineages recovered from the SNP analysis, and which morphological characters provide relatively stable diagnostic evidence for these lineages? Through these analyses, we aimed to establish a clearer phylogenetic framework for *Blastus*, reassess the taxonomic value of the traditional morphological groupings and their associated characters, and provide a foundation for further taxonomic evaluation of uncertain species and varieties.

## 2. Materials and Methods

### 2.1. Materials

#### 2.1.1. Materials for Morphological Study

The morphological study was based on 2174 herbarium specimens of *Blastus*, including 74 type specimens, together with 2048 digital images. Fresh material collected during field investigations and living plants cultivated in the greenhouse were also examined. Taxon identification and morphological comparisons followed primarily the treatment of *Blastus* in *Flora Reipublicae Popularis Sinicae* [16]. Images of type specimens were obtained mainly from JSTOR Global Plants and the online database of the Herbarium of the Institute of Botany, Chinese Academy of Sciences (PE). Additional type materials were examined directly or through photographs obtained from the Herbarium of the Kunming Institute of Botany, Chinese Academy of Sciences (KUN), the Herbarium of South China Botanical Garden, Chinese Academy of Sciences (IBSC), and the Hong Kong Herbarium (HK). Images and records of specimens other than types were obtained primarily from PE, the Chinese Virtual Herbarium (CVH), the Chinese Field Herbarium (CFH), the National Specimen Information Infrastructure (NSII), the Herbarium of Guangxi Institute of Botany (IBK), and the Herbarium of Xishuangbanna Tropical Botanical Garden (HITBC).

**Figure 1:**
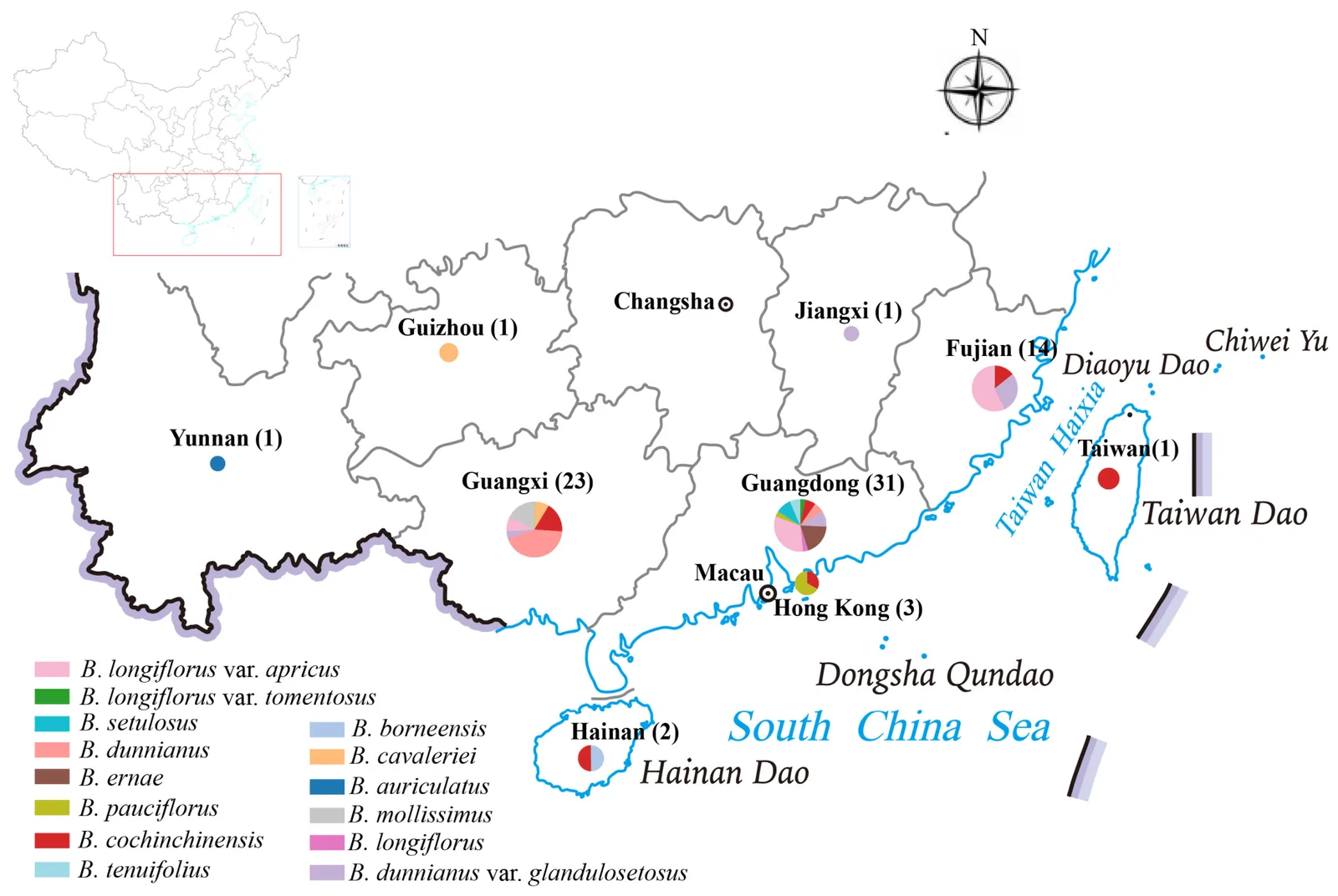
Geographic distribution and taxonomic composition of the *Blastus* accessions sampled in China. The pie charts indicate the number and taxonomic composition of the accessions from each province or region.

Specimen records from these herbaria and online databases were used to guide field sampling, which focused primarily on type localities and the main distribution areas of the recognized taxa. Following morphological identification, 81 samples of *Blastus*, representing 11 species and three varieties, were included in the broadly sampled ITS analysis. Representative samples were subsequently selected for the expanded nrDNA, plastome, and genomic SNP analyses. Figure 1 summarizes the geographic distribution and taxonomic composition of the samples collected in China. The three accessions from Vietnam and one sample from Borneo are listed in Table S1 but are not shown in the figure.

#### 2.1.2. Materials for Molecular Phylogenetic Analyses

The ITS dataset comprised all 81 field-collected accessions of *Blastus* and 12 accessions of related taxa, giving a total of 93 accessions. For the expanded nrDNA and plastome analyses, 16 representative *Blastus* accessions and all 12 related taxa were selected for genome sequencing. The 16 representative *Blastus* accessions were selected preferentially to include accessions from type localities, individuals with typical morphology, and taxa representing the principal morphological groups within the genus. The 12 related taxa represented genera previously reported as closely related to *Blastus*, including *Phyllagathis*, *Fordiophyton*, *Oxyspora*, and *Barthea*. Two more distantly related taxa, *Dissochaeta barbata* and *Pseudodissochaeta lanceata*, were included as outgroups in the ITS, nrDNA, and plastome phylogenetic analyses.

The genomic SNP dataset comprised 20 accessions, including the 16 representative *Blastus* accessions and four related taxa used as outgroups: *Fordiophyton faberi* 334, *Phyllagathis cavaleriei* 375, *Barthea barthei* 406, and *Phyllagathis rotundifolia* 699. These four taxa were selected from among the 12 related taxa included in the ITS, nrDNA, and plastome datasets. The SNP analysis was designed to resolve relationships among the principal intrageneric lineages of *Blastus* and to test the monophyly of the genus. Accordingly, these four representative related taxa were used as outgroups to root the SNP phylogeny and evaluate the monophyly of *Blastus* within the present sampling framework, while keeping the SNP matrix focused on relationships within *Blastus*.

### 2.2. Methods

#### 2.2.1. Morphological Analysis

Fresh material of *Blastus* collected during field investigations was photographed in natural habitats, and information on plant habit, habitat, and diagnostically relevant morphological characters was recorded. Flowers were collected from flowering individuals, dissected, and photographed. The principal characters examined included plant habit, the indumentum of young branchlets, leaf shape, leaf surface features, venation, the distribution of yellow glands on abaxial leaf surfaces, the presence or absence of petioles, inflorescence position and type, floral morphology, calyx lobe morphology, petal morphology, and anther and filament morphology. Particular attention was given to inflorescence position and type, indumentum, calyx lobe and petal morphology, and the distribution of yellow glands on the abaxial leaf surfaces during comparisons among taxa.

Quantitative measurements were conducted using procedures adapted to the morphology of *Blastus*. For each individual, three mature leaves were randomly selected from each of the upper, middle, and lower portions of the plant, giving a total of nine leaves per individual. Leaf length and width were measured in centimeters, and the measurements were averaged for each individual. For each flowering individual, three inflorescences were randomly selected from each plant, and three flowers were measured, resulting in a total of nine flowers measured per individual. Calyx lobe, petal, and anther lengths were measured in millimeters, and the measurements were averaged for each individual.

Taxon identification and morphological comparisons were based on an integrated examination of material collected in the field, herbarium specimens, images of type specimens, living plants, and the online resources described in Section 2.1.1. Leaf shape and venation, indumentum, the distribution of yellow glands, calyx lobe morphology, and floral structure were compared with the corresponding type material and published taxonomic descriptions to identify the sampled *Blastus* accessions.

#### 2.2.2. DNA Extraction, ITS Amplification, and Genome Sequencing

Total genomic DNA was extracted using the TIANGEN Plant Genomic DNA Kit (DP305; TIANGEN Biotech, Beijing, China). DNA integrity and concentration were assessed by agarose gel electrophoresis and Qubit fluorometric quantification using a Qubit 4 Fluorometer (Invitrogen, Thermo Fisher Scientific, Waltham, MA, USA), respectively. Two complementary nuclear ribosomal datasets were constructed using different sampling strategies. The broadly sampled ITS dataset was designed to maximize taxonomic and intraspecific representation, whereas the expanded nrDNA cistron dataset included fewer representative accessions but covered a larger portion of the cistron, including 18S, ITS1, 5.8S, ITS2, and 28S. For the broadly sampled dataset, the ITS region was amplified by PCR using the primers ITS-17SE and ITS-26SE. The PCR products were examined by agarose gel electrophoresis, purified, and subjected to Sanger sequencing by Shanghai Invitrogen Biotechnology Co., Ltd. (Guangzhou Branch, Guangzhou, China). Low coverage genome resequencing was then performed for the 16 representative *Blastus* accessions and 12 related taxa. After quality control, reads from all 28 accessions were used to assemble expanded nrDNA cistrons and plastomes.

Genomic SNP calling and phylogenetic analysis based on SNPs were conducted for the 16 representative *Blastus* accessions and four related taxa. For these accessions, resequencing reads were mapped to the chromosome-level reference genome of *Melastoma dodecandrum* (PRJCA005299) for SNP detection.

#### 2.2.3. ITS Sequence Processing

The broadly sampled ITS dataset comprised 93 accessions, including 81 *Blastus* accessions collected in the field and 12 accessions of related taxa. The ITS sequences generated by Sanger sequencing were assembled and manually checked using SeqMan Pro v17.0. The resulting consensus sequences were imported into Geneious Prime v2025.0.3 for additional inspection and editing. Sequence alignment, trimming, and phylogenetic analyses followed the workflow described in Section 2.2.6.

To reduce redundancy among tree terminals, identical ITS sequences within *Blastus* were collapsed, and one representative sequence was retained for each set of identical sequences. The reduced dataset used in the main phylogenetic analysis therefore contained 26 representative *Blastus* ITS sequences and 12 related taxa, for a total of 38 terminals.

#### 2.2.4. nrDNA Assembly and Analysis

The expanded nrDNA cistron dataset comprised 28 representative accessions, including 16 representative *Blastus* accessions and 12 related taxa. After quality control of the low coverage genome sequencing data, clean reads were used for nrDNA assembly. The nrDNA sequences were assembled using GetOrganelle v1.7.7.1, with the nrDNA sequence of *Wisteria floribunda* used as a reference to facilitate the identification and extraction of the target sequences [25]. When necessary, SPAdes v3.15.5 was used for additional assembly of the target regions [26]. The resulting nrDNA assemblies were imported into Geneious Prime for manual inspection and annotation [27]. The 18S, ITS1, 5.8S, ITS2, and 28S regions were identified and extracted together to construct the expanded nrDNA cistron dataset. This dataset included fewer representative accessions than the broadly sampled ITS dataset but provided greater nuclear ribosomal sequence coverage for resolving relationships among the principal lineages.

#### 2.2.5. Plastome Assembly and Analysis

Plastomes were assembled from the low coverage genome sequencing data using GetOrganelle [25]. The assembly graphs were visualized and inspected using Bandage to evaluate the completeness and structure of each plastome assembly [28]. The assembled plastomes were annotated in Geneious Prime and manually corrected through comparison with published plastomes of closely related taxa [27]. The annotated plastome sequences were subsequently used for phylogenetic analysis.

#### 2.2.6. Phylogenetic Analyses of the ITS, nrDNA, and Plastome Datasets

The broadly sampled ITS dataset, the expanded nrDNA cistron dataset, and the plastome dataset were analyzed separately using the same general phylogenetic workflow. Sequences in each dataset were aligned using MAFFT implemented in Geneious Prime v2025.0.3 [29]. Each alignment was inspected manually, and poorly aligned regions and regions containing extensive missing data were removed using trimAl through PhyloSuite v1.2.3 [30,31].

Maximum likelihood analyses were performed using IQ-TREE through PhyloSuite [32,33]. For each dataset, ModelFinder was used to select the best fitting nucleotide substitution model according to the Bayesian information criterion [34]. Branch support was assessed using 1000 ultrafast bootstrap replicates [35]. Bayesian inference analyses were conducted using MrBayes through PhyloSuite. The topologies obtained from the maximum likelihood and Bayesian analyses were compared, and ultrafast bootstrap support values and Bayesian posterior probabilities were mapped onto the maximum likelihood trees and presented as UFBS/PP. Nodes not recovered in the BI topology were indicated by dashes. *Dissochaeta barbata* and *Pseudodissochaeta lanceata* were used as outgroups for the ITS, nrDNA, and plastome analyses.

#### 2.2.7. SNP Detection and Phylogenetic Analysis

Genomic SNP analysis was conducted for 20 accessions, comprising 16 representative *Blastus* accessions and four closely related taxa used as outgroups. The chromosome-level genome assembly of *Melastoma dodecandrum*, assembled using Hi-C data and available under project accession PRJCA005299, was used as the reference genome.

To maintain a consistent analytical framework across samples, all 20 accessions were processed using the same reference genome and variant calling workflow. The reference genome was indexed using BWA v0.7.17-r1188 and SAMtools v1.21, and a sequence dictionary was generated using GATK v4.6.2.0. Clean reads from paired end sequencing were mapped to the reference genome using BWA-MEM [36]. The resulting alignments were sorted and indexed using SAMtools [37]. Before variant calling, BAM-file integrity, reference consistency, index files, and read group information were checked to ensure compatibility with the GATK workflow.

Variant calling was performed using the GVCF workflow for joint genotyping implemented in GATK v4.6.2.0 [38,39]. HaplotypeCaller was used to generate an individual gVCF file for each accession with the ‘-ERC GVCF’ option. The individual gVCF files were then combined using CombineGVCFs, after which joint genotyping was subsequently performed using GenotypeGVCFs to generate a raw VCF file containing all samples. SNPs were extracted using SelectVariants. Hard filtering was performed using VariantFiltration with the following thresholds: ‘QD < 2.0’, ‘FS > 60.0’, ‘MQ < 40.0’, ‘SOR > 3.0’, ‘MQRankSum < −12.5’, and ‘ReadPosRankSum < −8.0’. Only biallelic SNPs marked as PASS were retained.

The filtered VCF file was converted into a nucleotide matrix for phylogenetic analysis. Homozygous reference genotypes were represented by the reference nucleotide, homozygous alternative genotypes by the alternative nucleotide, heterozygous genotypes by the corresponding IUPAC ambiguity codes, and missing genotypes by ‘N’. To reduce the influence of missing and ambiguous data, only sites at which at least 18 of the 20 accessions had unambiguous A, C, G, or T states were retained. The final matrix contained 122,382 SNPs, of which 27,657 were parsimony-informative, accounting for 22.60% of the total. A maximum likelihood tree was reconstructed from the filtered SNP matrix using IQ-TREE v2 [33]. Because the matrix contained only variable sites, the ‘GTR+ASC’ model was applied to correct for ascertainment bias [40]. Branch support was evaluated using 1000 ultrafast bootstrap replicates and 1000 SH-aLRT replicates [35]. The analysis was run with the parameters ‘-st DNA -m GTR+ASC -B 1000 -alrt 1000 -T 32’. The maximum likelihood tree was rooted on the branch separating the four designated outgroups, *Fordiophyton faberi* 334, *Phyllagathis cavaleriei* 375, *Barthea barthei* 406, and *Phyllagathis rotundifolia* 699, from the *Blastus* clade.

## 3. Results

### 3.1. Morphological Characteristics and Comparisons

#### 3.1.1. General Morphological Characteristics of Blastus

Field observations indicated that species of *Blastus* are predominantly shrubs. The stems are cylindrical and are often covered with minute glandular trichomes. The leaves are thinly papery to membranous, entire or shallowly serrulate, with 3–5(–7) basal veins and subparallel lateral veins. Petiole development varies within the genus. For example, *B. auriculatus* is characterized by auriculate leaf bases and sessile or subsessile leaves, whereas most other species have distinct petioles. The bracts are small and caducous, and the flowers are usually tetramerous. The calyx is narrowly funnelform, campanulate to funnelform, or cylindrical and often covered with minute glands. The calyx lobes are small and shortly apiculate. The petals are white, pink, or pale purple, ovate, or oblong and usually acuminate at the apex.

**Figure 2:**
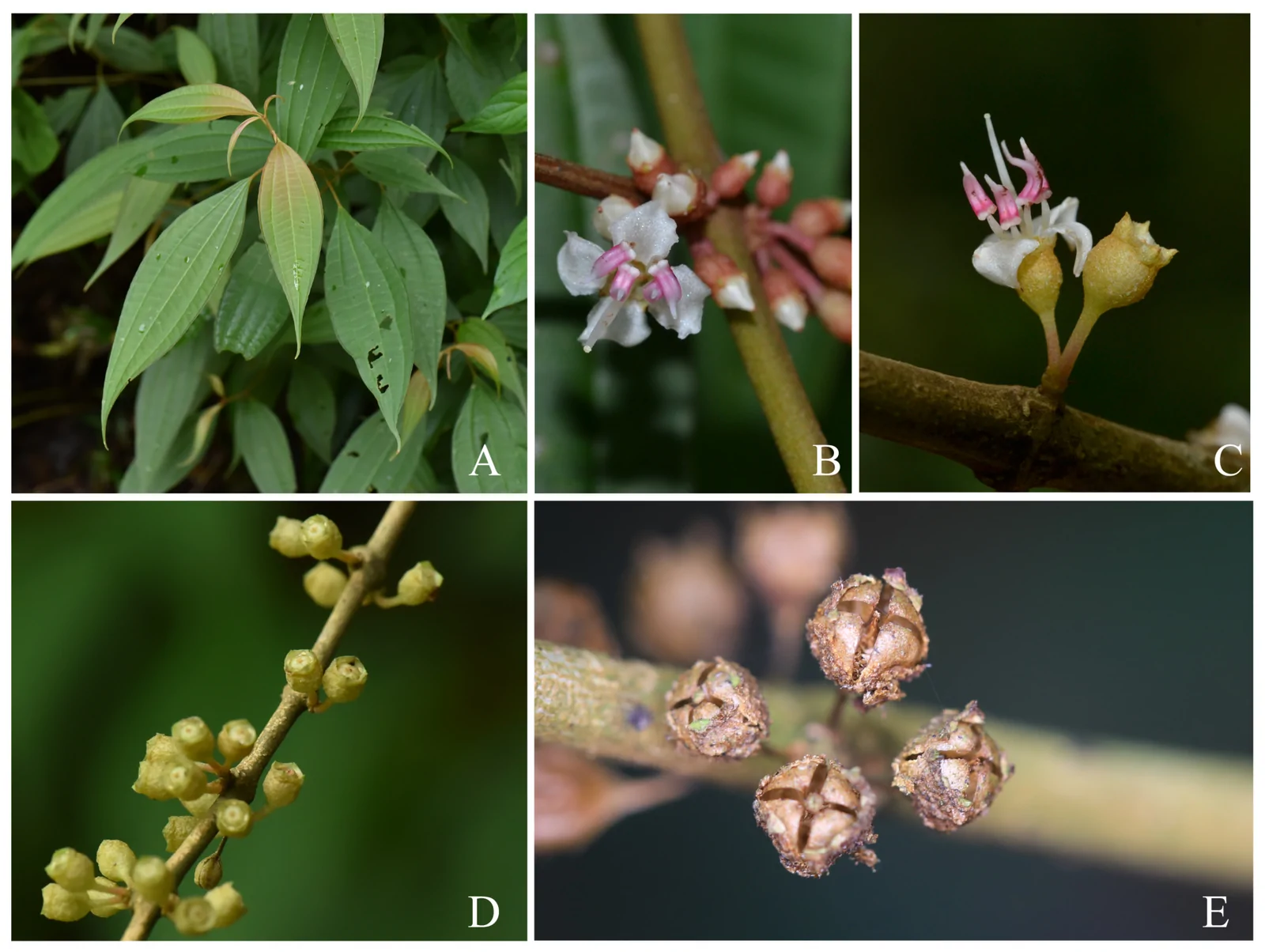
Morphological characteristics of *Blastus cochinchinensis*. (**A**) Leaf showing the basal venation and serrulate margin; (**B**) flower in frontal view, showing the 4-merous corolla; (**C**) flower in lateral view, showing the hypanthium and calyx lobes; (**D**) immature fruit showing the persistent calyx lobes; (**E**) mature fruit. All photographs were taken by the authors.

These characters represent the general morphological range of *Blastus* but provide limited value for distinguishing closely related taxa. Comparative observations indicated that inflorescence position, the distribution of yellow glands on the abaxial leaf surface, and the shape and degree of reflexion of the calyx lobes were more informative for distinguishing the principal morphological groups. By contrast, indumentum characters varied considerably within these groups. For comparison, the examined taxa are described below according to the two broad morphological assemblages recognized by Diels, namely the axillary inflorescence group and the terminal inflorescence group. Within the terminal inflorescence group, the *B. pauciflorus* group and the *B. cavaleriei* group are further compared as two informal working units. The principal diagnostic characters of each taxon are summarized in Table S2, and representative morphological features are illustrated in Figure 2.

#### 3.1.2. Comparison Between the Axillary Inflorescence Group and the Terminal Inflorescence Group

**Figure 3:**
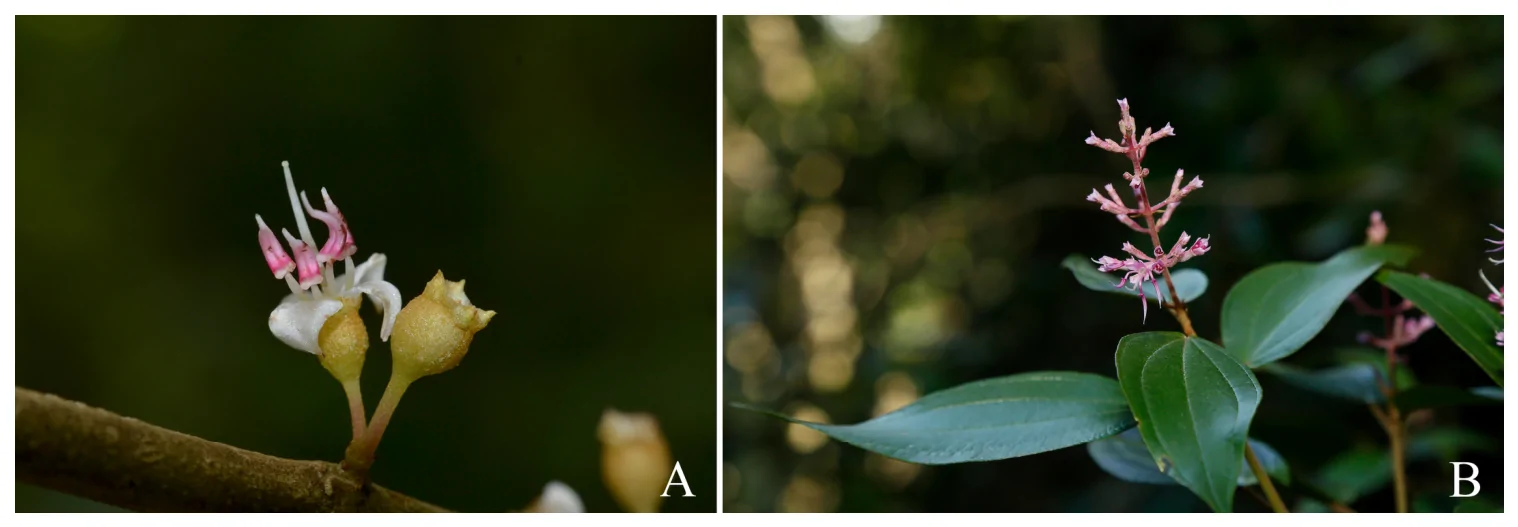
Inflorescence position and type in *Blastus*. (**A**) *Blastus cochinchinensis*, with axillary inflorescences; (**B**) *Blastus pauciflorus*, with terminal inflorescences. All photographs were taken by the authors.

Based primarily on inflorescence position and architecture, species of *Blastus* can be divided into two major morphological groups. One group is characterized by axillary inflorescences borne in the leaf axils or on leafless portions of the stems, whereas the other is characterized by terminal inflorescences. These two morphological groups broadly correspond to the two descriptive assemblages proposed by Diels [23], here referred to as the axillary inflorescence group and the terminal inflorescence group, respectively (Table S2, Figure 3).

Six taxa of the axillary inflorescence group were examined in this study: *B. cochinchinensis*, *B. mollissimus*, *B. auriculatus*, *B. tenuifolius*, *B. setulosus*, and *B. borneensis*. With the exception of *B. borneensis*, which possesses compound cymose inflorescences, the remaining taxa possess umbellate or umbelliform cymes. These inflorescences are borne in the leaf axils or on leafless portions of the stems and have short or nearly absent peduncles (Figures S1–S6). Although *B. mollissimus* is distinctive in its indumentum, it agrees with the general morphology of the axillary inflorescence group in having subparallel lateral veins, petiolate leaves, axillary umbelliform cymes, and tetramerous flowers.

**Figure 4:**
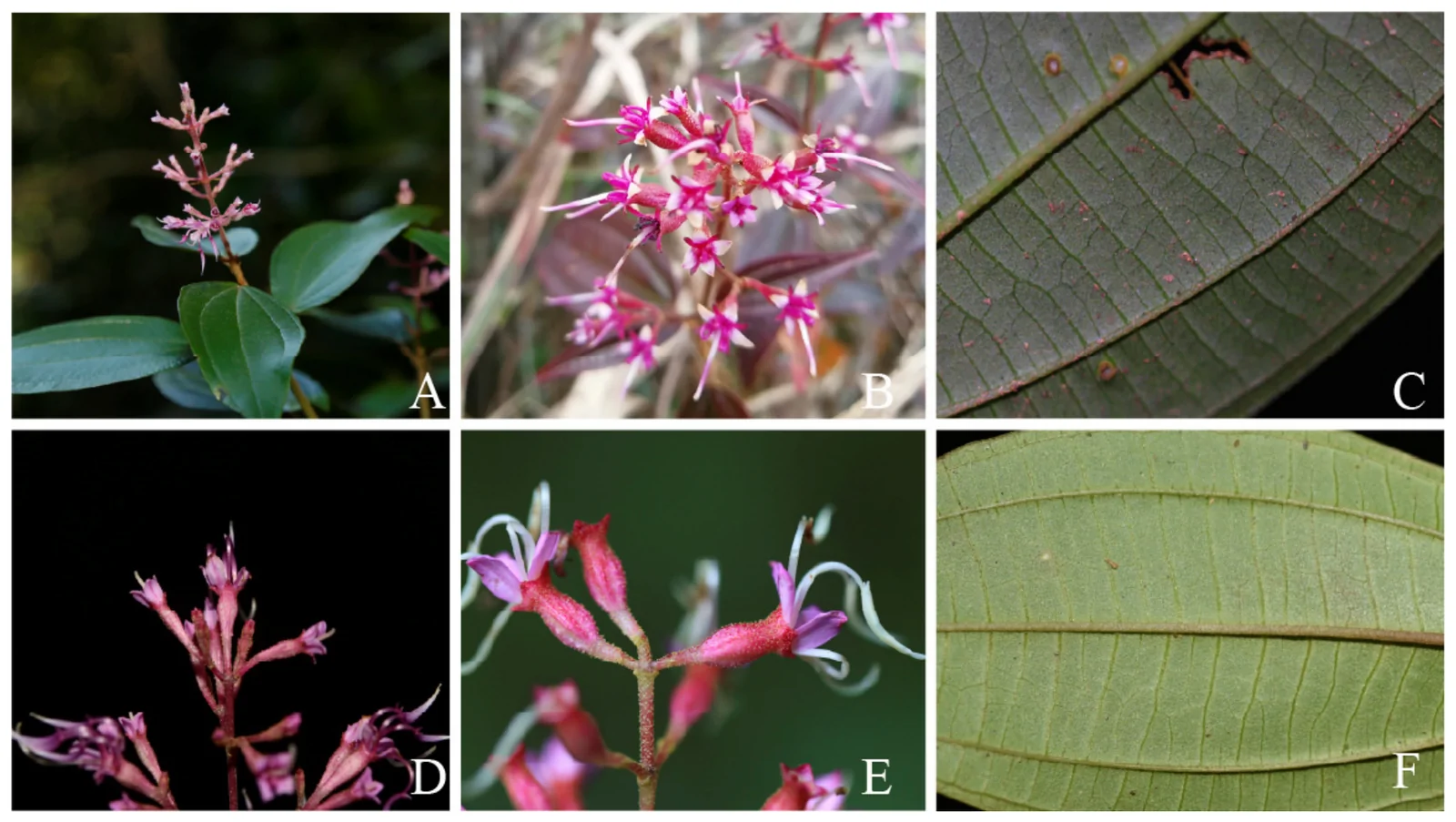
Comparison of calyx-lobe morphology and the distribution of yellow glands on the abaxial leaf surface in the *Blastus pauciflorus* group. (**A**) *B. pauciflorus*, showing shortly triangular calyx lobes; (**B**) *B. longiflorus* var. *apricus*, showing linear-triangular calyx lobes; (**C**) abaxial leaf surface of *B. longiflorus* var. *apricus*, showing scattered yellow glands; (**D**) *B. longiflorus*, showing shortly triangular calyx lobes; (**E**) *B. ernae*, showing broadly triangular calyx lobes; (**F**) abaxial leaf surface of *B. longiflorus*, showing scattered yellow glands. Photograph (**A**) courtesy of the Hong Kong Herbarium; photographs (**B**–**F**) were taken by the authors.

**Figure 5:**
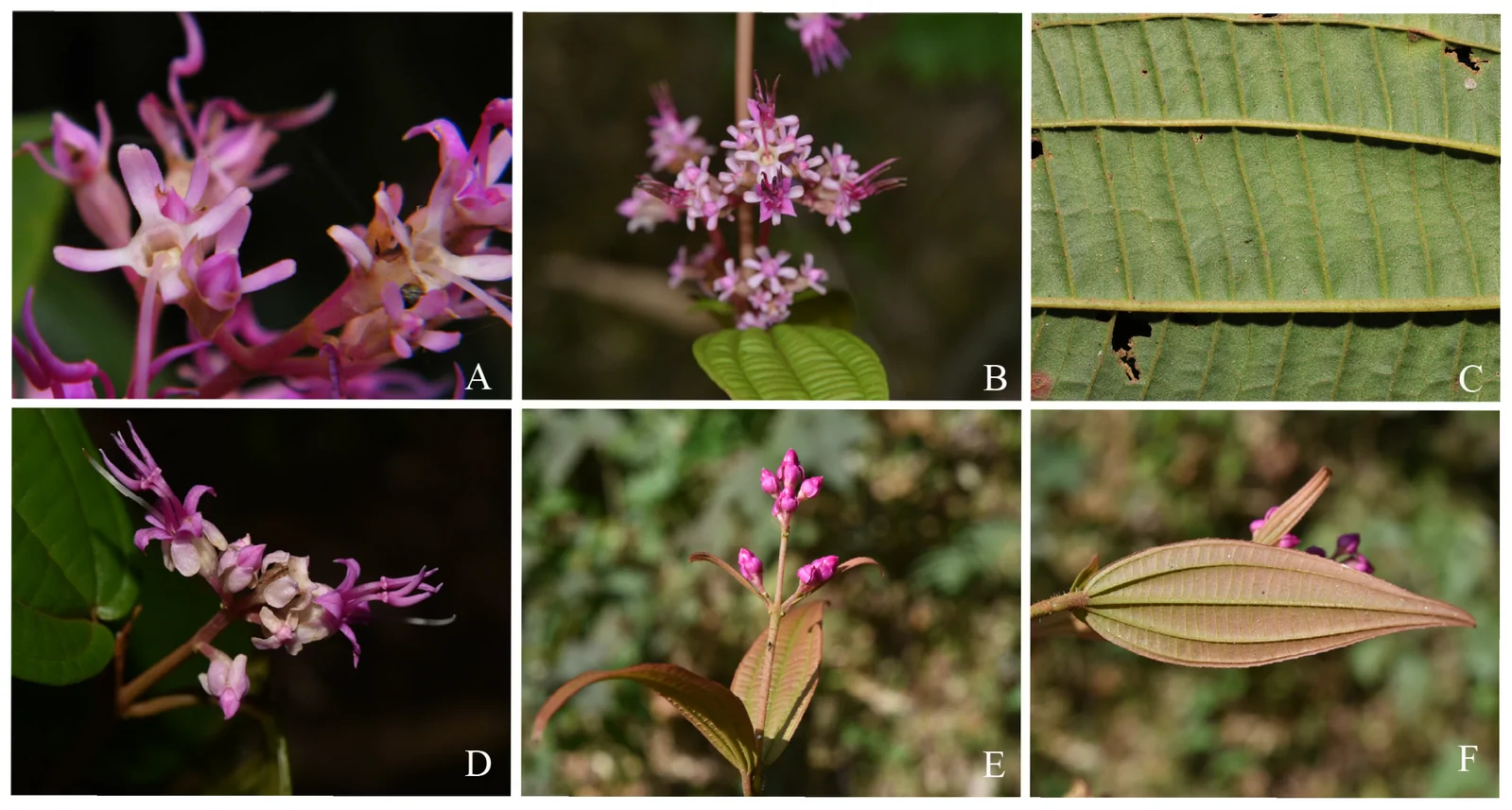
Comparison of calyx-lobe morphology and the distribution of yellow glands on the abaxial leaf surface in the *Blastus cavaleriei* group. (**A**) *B. cavaleriei*, showing spatulate, non-reflexed calyx lobes; (**B**) *B. cavaleriei* var. *tomentosus*, showing spatulate, non-reflexed calyx lobes; (**C**) abaxial leaf surface of *B. dunnianus*, showing yellow glands distributed mainly along the veins; (**D**) *B. dunnianus*, showing ovate, reflexed calyx lobes; (**E**) *B. dunnianus* var. *glandulosetosus*, showing ovate, reflexed calyx lobes; (**F**) abaxial leaf surface of *B. dunnianus* var. *glandulosetosus*, showing yellow glands distributed mainly along the veins. All photographs were taken by the authors.

Eight taxa of the terminal inflorescence group were examined: *B. longiflorus*, *B. longiflorus* var. *apricus*, *B. ernae*, *B. pauciflorus*, *B. cavaleriei*, *B. cavaleriei* var. *tomentosus*, *B. dunnianus*, and *B. dunnianus* var. *glandulosetosus*. In contrast to the axillary inflorescence group, members of the terminal inflorescence group possess terminal paniculate inflorescences, peduncles usually longer than 4 cm, and predominantly pink to red corollas. For morphological comparison, the sampled taxa of the terminal inflorescence group were provisionally divided into two informal groups, the *B. pauciflorus* group and the *B. cavaleriei* group. These groups were distinguished primarily by the presence and distribution of yellow glands on the abaxial leaf surface and by the shape and degree of reflexion of the calyx lobes (Table S2). The *B. pauciflorus* group comprises *B. pauciflorus*, *B. longiflorus*, *B. longiflorus* var. *apricus*, and *B. ernae*. The *B. cavaleriei* group comprises *B. cavaleriei*, *B. cavaleriei* var. *tomentosus*, *B. dunnianus*, and *B. dunnianus* var. *glandulosetosus* (Figure 4 and Figure 5).

#### 3.1.3. Morphological Comparison of the *B. pauciflorus* and the *B. cavaleriei* Groups

To assess morphological differentiation within the terminal inflorescence group, we compared the principal characters of the *B. pauciflorus* and *B. cavaleriei* groups, focusing on leaf base morphology, the indumentum of vegetative and reproductive organs, the morphology of the calyx tube and lobes, petal characters, and anther morphology (Tables S3 and S4).

##### The *B*. *pauciflorus* Group

The *B. pauciflorus* group comprised four taxa: *B. pauciflorus*, *B. longiflorus*, *B. longiflorus* var. *apricus*, and *B. ernae*. *B. pauciflorus* was distinguished from the other three taxa by its markedly shorter anthers, which were approximately 3 mm long and had obtuse, slightly divergent bases. By comparison, *B*. *longiflorus*, *B. longiflorus* var. *apricus*, and *B. ernae* had anthers approximately 8 mm long with hornlike bases (Table S3).

The latter three taxa differed mainly in calyx lobe shape and indumentum. *B*. *longiflorus* var. *apricus* had linear triangular calyx lobes, *B. longiflorus* had shortly triangular calyx lobes, and *B. ernae* had broadly triangular calyx lobes and calyx tubes bearing stipitate glandular hairs. Field observations indicated that the development of stipitate glandular hairs in *B. ernae* varied among individuals. Calyx lobe morphology also exhibited continuous variation among these three taxa (Figures S7–S10).

##### The *B*. *cavaleriei* Group

The *B. cavaleriei* group comprised *B. cavaleriei*, *B. cavaleriei* var. *tomentosus*, *B. dunnianus*, and *B. dunnianus* var. *glandulosetosus*. All four taxa had yellow glands that were confined to the veins on the abaxial leaf surfaces but differed in calyx-lobe morphology, anther-base morphology, and the type and distribution of indumentum (Table S4).

*Blastus cavaleriei* and *B. cavaleriei* var. *tomentosus* both had spatulate calyx lobes that were not reflexed. *Blastus cavaleriei* was characterized primarily by ferruginous puberulence and sparse glands, whereas *B. cavaleriei* var. *tomentosus* bore spreading glandular hairs intermixed with puberulence on the young branchlets, petioles, abaxial leaf veins, and inflorescences. The anther bases were slightly swollen in both taxa, but the swelling was more pronounced in *B. cavaleriei* var. *tomentosus*. Field observations showed that the abundance and development of glandular hairs in this variety varied among individuals. Some specimens also had calyx lobes intermediate between spatulate and ovate forms (Figures S11 and S12).

*Blastus dunnianus* and *B. dunnianus* var. *glandulosetosus* both had ovate, reflexed calyx lobes and anthers with a small, inconspicuous tubercle at the base. *Blastus dunnianus* was characterized mainly by ferruginous puberulence and sparse glands, whereas *B. dunnianus* var. *glandulosetosus* bore conspicuous glandular setae on the young branchlets, petioles, abaxial leaf veins, and inflorescences. The abundance and development of these glandular setae varied among individuals collected from different localities, and some variation in calyx-lobe morphology was also observed among populations of *B. dunnianus* (Figures S13 and S14).

Overall, the two informal groups were differentiated most consistently by the distribution of yellow glands on the abaxial leaf surfaces and by calyx-lobe morphology. In the *B. pauciflorus* group, the yellow glands were generally extended beyond the principal veins, and the calyx lobes ranged from linear triangular to broadly triangular. In the *B. cavaleriei* group, the yellow glands were mainly confined to the veins, and the calyx lobes were typically spatulate or ovate. By contrast, indumentum characters, particularly the abundance and development of glandular hairs and setae, varied more extensively among individuals and populations.

### 3.2. Phylogenetic Analyses Based on Nuclear Markers

#### 3.2.1. ITS Phylogeny

**Figure 6:**
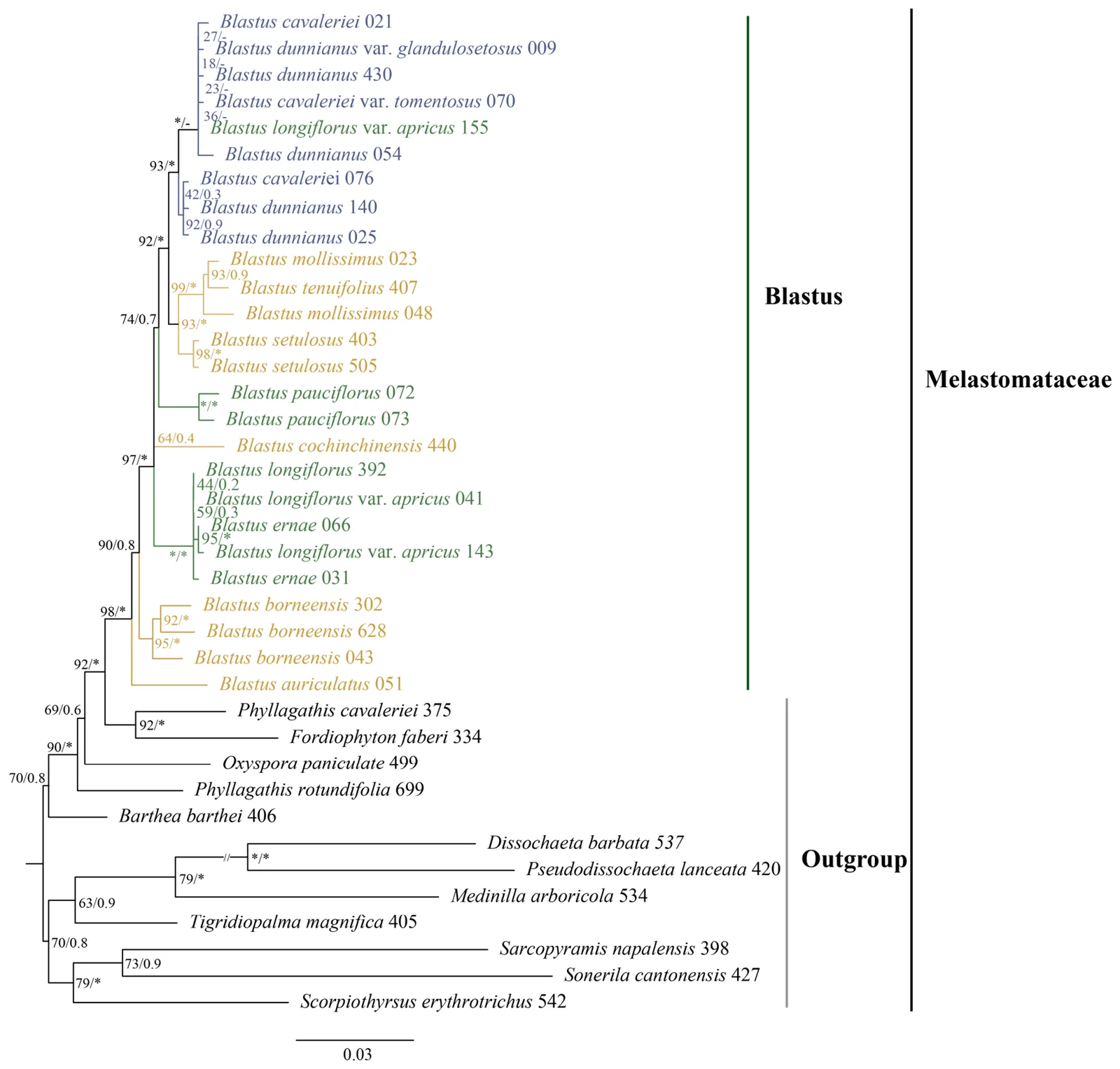
Maximum likelihood phylogenetic tree of *Blastus* and related taxa inferred from the representative ITS dataset. Values adjacent to the branches indicate ultrafast bootstrap support and Bayesian posterior probability (UFBS/PP), respectively. Asterisks indicate maximum support (UFBS = 100 or PP = 1.00), and dashes indicate that the corresponding node was not recovered in the Bayesian inference tree. Colors indicate the morphological assignments of the sampled taxa: yellow, the axillary inflorescence group; green, the *B. pauciflorus* group; and blue, the *B. cavaleriei* group. The scale bar represents the expected number of substitutions per site. Numbers following taxon names indicate sample accession numbers used in this study.

ITS sequences were obtained from 81 accessions of *Blastus* and 12 accessions representing related taxa. The phylogenetic tree inferred from the complete dataset of 93 accessions is presented in Figure S15. Because some *Blastus* accessions, including accessions collected from different localities, shared identical ITS sequences, one representative sequence was retained for each set of identical sequences to reduce redundancy among tree terminals. The reduced dataset comprised 26 representative *Blastus* sequences and 12 sequences from related taxa, for a total of 38 terminals. After alignment and trimming, the final matrix was 782 bp in length; contained 311 variable sites, accounting for 39.77% of the matrix; and had 182 parsimony-informative sites, accounting for 23.27% (Table S5). The maximum likelihood and Bayesian inference analyses yielded broadly congruent topologies for the principal relationships. The following description is therefore based primarily on the maximum likelihood tree shown in Figure 6.

The sampled accessions of *Blastus* formed a strongly supported monophyletic lineage (UFBS = 98, PP = 1.00). However, neither the axillary inflorescence group nor the two informal morphological groups within the terminal inflorescence group, the *B. pauciflorus* group and the *B. cavaleriei* group, was recovered as monophyletic.

Within *Blastus*, *B. auriculatus* was resolved as sister to all remaining accessions. The three accessions of *B. borneensis* formed a strongly supported clade (UFBS = 95, PP = 1.00) that was sister to the clade comprising all remaining taxa except *B. auriculatus*. The remaining members of the axillary inflorescence group were distributed across several positions within the genus. Accessions of *B. mollissimus*, *B. tenuifolius*, and *B. setulosus* formed an internal clade that was sister to the clade containing members of the *B. cavaleriei* group (UFBS = 92, PP = 1.00). Within this clade, however, *B. mollissimus* and *B. tenuifolius* did not form separate exclusive lineages. *B*. *cochinchinensis* was resolved on a separate internal branch, but its placement received weak support (UFBS = 64, PP = 0.40).

The *B. pauciflorus* group was distributed across three positions in the ITS tree. The two accessions of *B. pauciflorus* formed a strongly supported clade (UFBS = 100, PP = 1.00). Most retained sequences of *B. longiflorus*, *B. longiflorus* var. *apricus*, and *B. ernae* formed another strongly supported clade (UFBS = 100, PP = 1.00). However, representatives of the three taxa were intermingled within this clade and did not form exclusive lineages corresponding to individual taxa. In addition, *B. longiflorus* var. *apricus* 155 was separated from the other accessions of this variety and was nested within the clade containing the *B. cavaleriei* group.

In both the maximum likelihood and Bayesian inference analyses, all retained sequences assigned to the *B. cavaleriei* group clustered together with *B. longiflorus* var. *apricus* 155 (UFBS = 93, PP = 1.00). Within this clade, accessions of *B. cavaleriei*, *B. cavaleriei* var. *tomentosus*, *B. dunnianus*, and *B. dunnianus* var. *glandulosetosus* were intermingled and several relationships near the tips received weak support. The *B. cavaleriei* group was therefore not recovered as monophyletic because the clade also contained *B. longiflorus* var. *apricus* 155. Moreover, none of its four constituent taxa formed an exclusive lineage.

Overall, the ITS analyses strongly supported the monophyly of the sampled *Blastus* accessions but provided limited resolution of relationships among closely related taxa. The resulting topology showed only partial correspondence with the morphological classification and did not support the monophyly of the axillary inflorescence group or either of the two informal groups within the terminal inflorescence group.

#### 3.2.2. nrDNA Phylogeny

To complement the broadly sampled ITS analysis and improve the resolution of relationships among the principal lineages of *Blastus*, an expanded nrDNA dataset was constructed using 16 representative *Blastus* accessions and 12 related taxa. The dataset comprised the 18S, ITS1, 5.8S, ITS2, and 28S regions, thereby providing greater nuclear ribosomal sequence coverage for the representative accessions.

**Figure 7:**
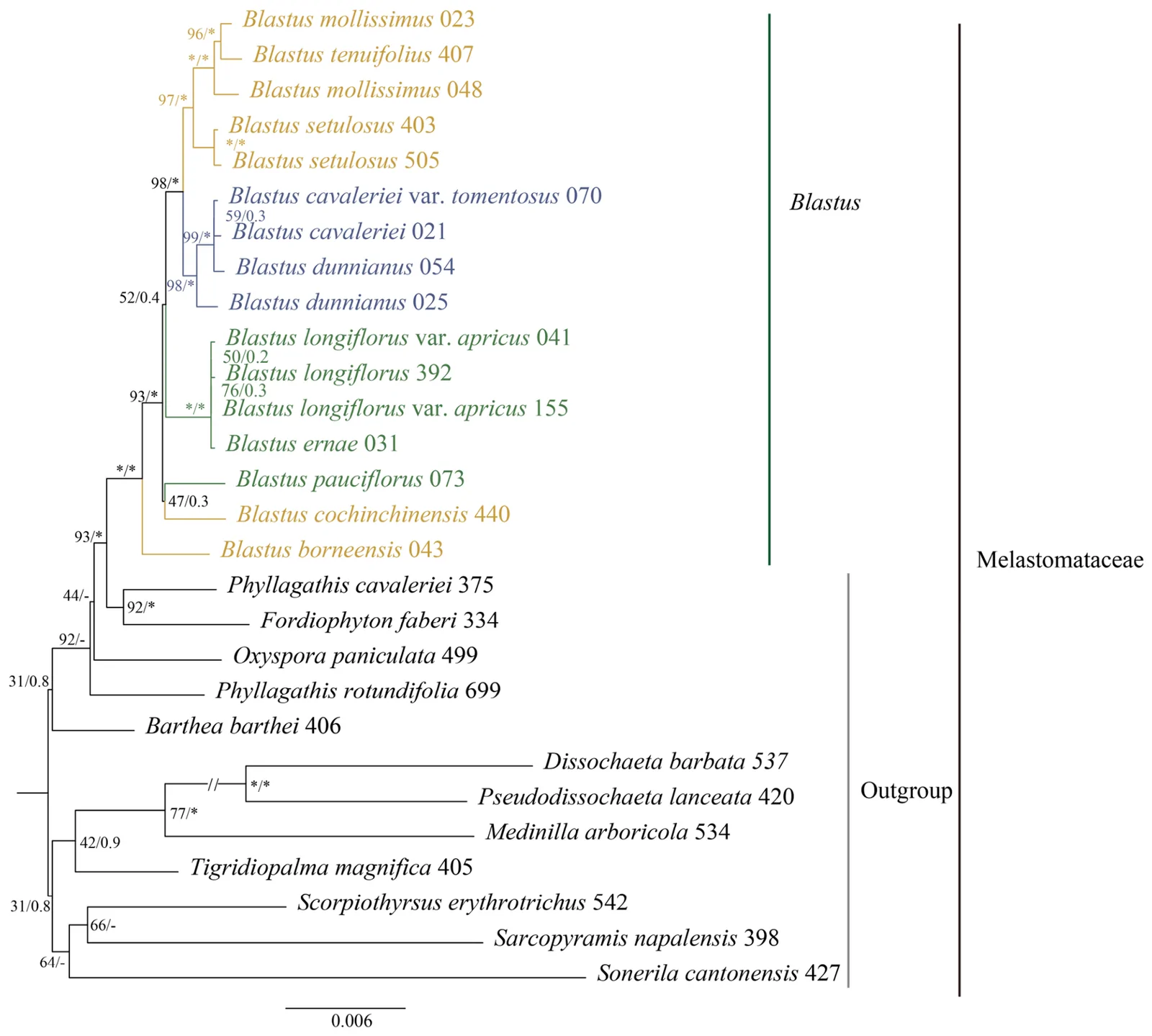
Maximum likelihood phylogenetic tree of *Blastus* and related taxa inferred from the nrDNA dataset. Values adjacent to the branches indicate ultrafast bootstrap support and Bayesian posterior probability (UFBS/PP), respectively. Asterisks indicate maximum support (UFBS = 100 or PP = 1.00), whereas dashes indicate that the corresponding node was not recovered in the Bayesian inference tree. Yellow, green, and blue denote the axillary inflorescence group, the *B. pauciflorus* group, and the *B. cavaleriei* group, respectively. The scale bar represents the expected number of substitutions per site. Numbers following taxon names indicate sample accession numbers used in this study.

After sequence alignment and trimming, the final matrix was 5840 bp, including 529 variable sites (9.06%) and 274 parsimony-informative sites (4.69%) (Table S5). The maximum likelihood and Bayesian inference analyses produced broadly congruent topologies. The following description is therefore based primarily on the maximum likelihood tree shown in Figure 7.

*Blastus* was recovered as a strongly supported monophyletic group (UFBS = 100, PP = 1.00). However, neither the sampled members of the axillary inflorescence group nor those of the *B. pauciflorus* group were recovered as monophyletic. In contrast, the sampled members of the *B. cavaleriei* group formed an exclusive clade.

Within *Blastus*, *B. borneensis* was resolved as sister to all remaining accessions. The other sampled members of the axillary inflorescence group were distributed across separate positions within the genus. *B*. *mollissimus*, *B. tenuifolius*, and *B. setulosus* formed a clade that was sister to the clade comprising the sampled members of the *B. cavaleriei* group. However, the accessions of *B. mollissimus* and *B. tenuifolius* did not form separate exclusive lineages. *B*. *cochinchinensis* was resolved separately from the other sampled members of the axillary inflorescence group (UFBS = 47, PP = 0.30).

The *B. pauciflorus* group was not recovered as monophyletic, as *B. pauciflorus* was placed as sister to *B. cochinchinensis* instead of clustering with the other members of the group. In contrast, *B. longiflorus*, *B. longiflorus* var. *apricus*, and *B. ernae* formed a strongly supported clade (UFBS = 100, PP = 1.00). Relationships within this clade received weak support, and the two accessions of *B. longiflorus* var. *apricus * did not form an exclusive lineage.

The sampled members of the *B. cavaleriei* group, namely *B. cavaleriei*, *B. cavaleriei* var. *tomentosus*, and *B. dunnianus*, formed an exclusive clade in both the maximum likelihood and Bayesian inference analyses. No member of the *B. pauciflorus* group was nested within this clade. However, relationships among the sampled taxa were not fully resolved. The sister relationship between *B. cavaleriei* and *B. cavaleriei* var. *tomentosus* received weak support (UFBS = 59, PP = 0.30), and the two accessions of *B. dunnianus* were not recovered as an exclusive lineage.

Compared with the ITS tree, the expanded nrDNA tree recovered the sampled members of the *B. cavaleriei* group as an exclusive clade and placed *B. longiflorus* var. *apricus* 155 with the other sample accessions of *B. longiflorus*, *B. longiflorus* var. *apricus*, and *B. ernae*. Nevertheless, neither the sampled members of the axillary inflorescence group nor those of the *B. pauciflorus* group were recovered as monophyletic, and several relationships among closely related taxa remained weakly supported.

#### 3.2.3. Plastome Phylogeny

**Figure 8:**
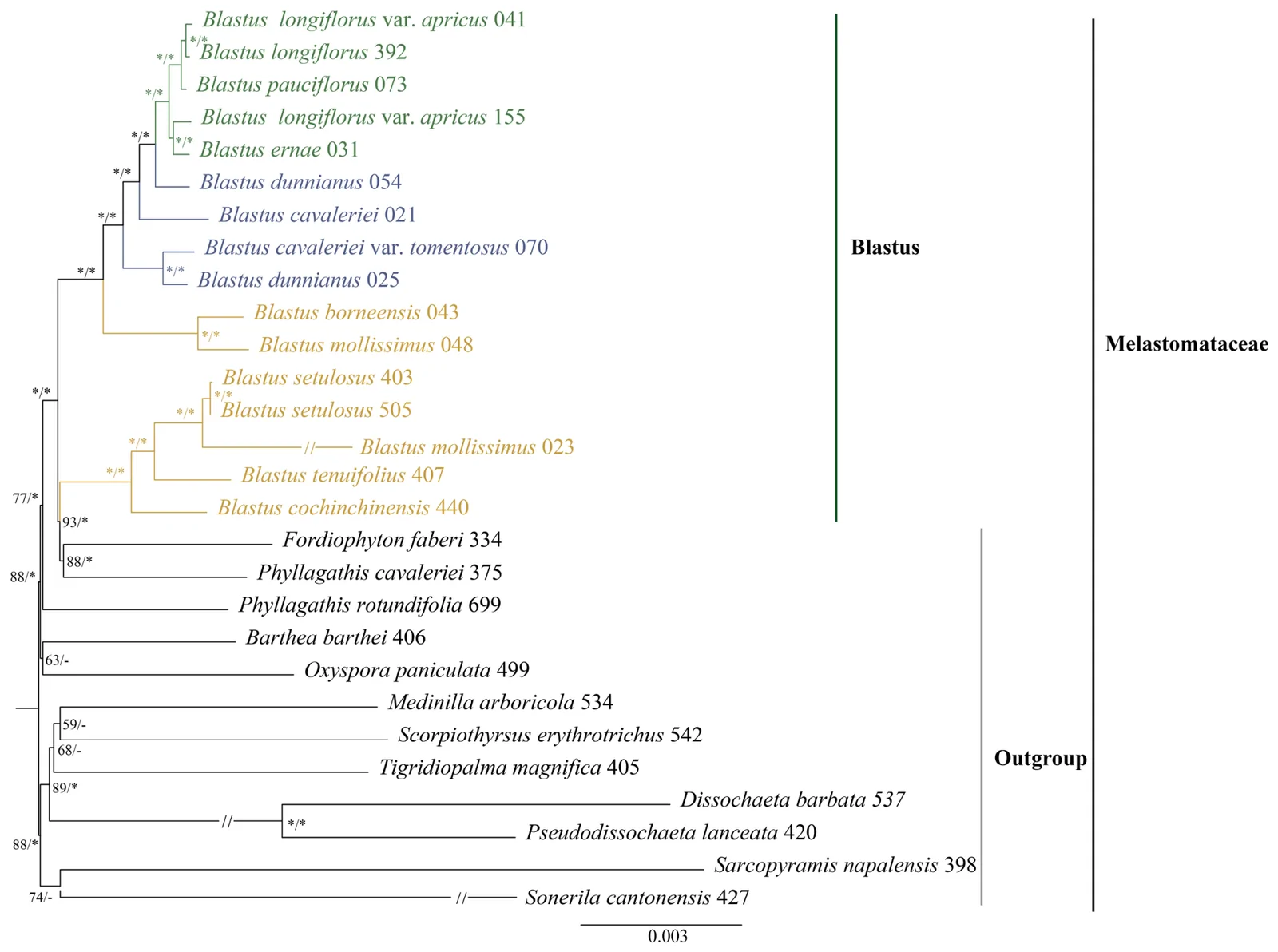
Maximum likelihood phylogeny of *Blastus* and related taxa inferred from the plastome dataset. Values adjacent to the branches indicate ultrafast bootstrap support and Bayesian posterior probability (UFBS/PP), respectively. Asterisks indicate maximum support for the corresponding measure (UFBS = 100 or PP = 1.00), and dashes indicate that the corresponding node was not recovered in the Bayesian inference tree. Orange, green, and blue denote the axillary inflorescence group, the *B. pauciflorus* group, and the *B. cavaleriei* group, respectively. The scale bar represents the expected number of substitutions per site. Numbers following taxon names indicate sample accession numbers used in this study.

The plastome dataset comprised 16 representative accessions of *Blastus* and 12 accessions of related taxa. After sequence alignment and trimming, the final matrix was 123,270 bp in length and contained 12,741 variable sites, accounting for 10.34% of the matrix, and 3450 parsimony informative sites, accounting for 2.80% (Table S5). The maximum likelihood and Bayesian inference analyses yielded broadly congruent topologies for the principal relationships. The following description is therefore based primarily on the maximum likelihood tree shown in Figure 8.

In contrast to the ITS and expanded nrDNA phylogenies, the plastome analysis did not recover the sampled *Blastus* accessions as monophyletic. Instead, these accessions were distributed across two major lineages. One lineage comprised all sampled members of the terminal inflorescence group together with *B. borneensis* 043 and *B. mollissimus* 048. The other lineage comprised the remaining sampled members of the axillary inflorescence group. This second lineage was recovered as more closely related to *Fordiophyton faberi* and *Phyllagathis cavaleriei* than to the other *Blastus* lineage.

Within the terminal inflorescence group, the sampled members of the *B. pauciflorus* group formed an exclusive lineage, whereas those of the *B. cavaleriei* group did not form an exclusive clade. The sampled members of the axillary inflorescence group were also divided between the two major lineages, and the two accessions of *B. mollissimus* were resolved in different positions. Overall, the plastome topology was markedly incongruent with both the nuclear phylogenies and showed only limited correspondence with the morphological classification.

#### 3.2.4. SNP Phylogeny

**Figure 9:**
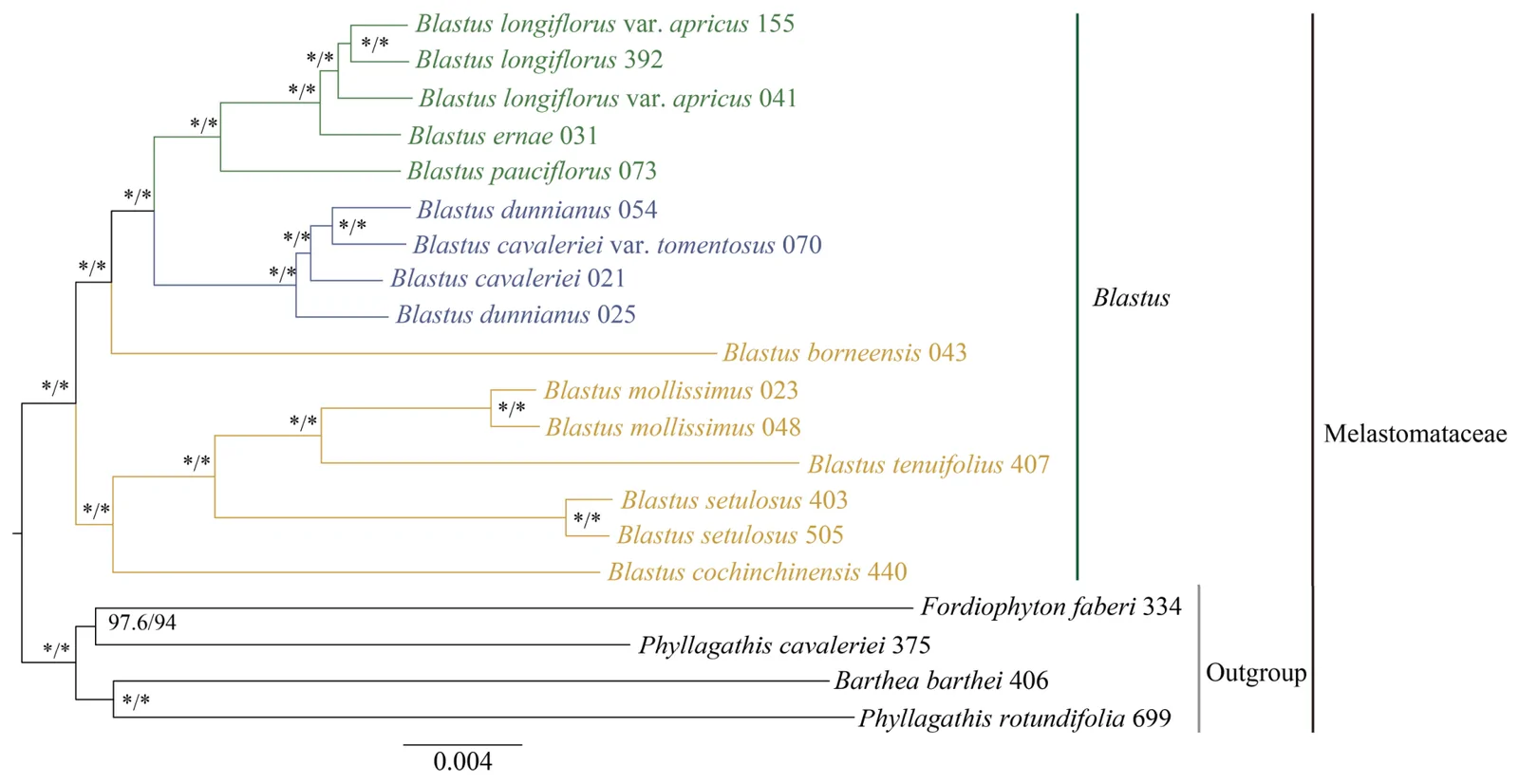
Maximum-likelihood phylogeny of 20 accessions inferred from the genomic SNP dataset, comprising 16 representative *Blastus* accessions and four closely related outgroup taxa. Values adjacent to the branches indicate SH-aLRT support and ultrafast bootstrap support (SH-aLRT/UFBS), each based on 1000 replicates. Green, blue, and orange indicate the *B. pauciflorus* group, the *B. cavaleriei* group, and the axillary inflorescence group, respectively. The scale bar represents the expected number of substitutions per site. Numbers following taxon names indicate sample accession numbers used in this study. Asterisks indicate maximum support for the corresponding measure (SH-aLRT = 100 or UFBS = 100).

The broadly sampled ITS and expanded nrDNA analyses both strongly supported the monophyly of the sampled *Blastus* accessions but provided limited resolution of relationships among closely related taxa within the genus. To further resolve the principal lineages within *Blastus* and test the monophyly of the sampled *Blastus* accessions, we analyzed genomic SNP data from 20 accessions, comprising 16 representative *Blastus* accessions and four related taxa used as outgroups. After quality filtering of variants and removal of sites with excessive missing or ambiguous states, the final SNP matrix contained 122,382 variable sites, of which 27,657 were parsimony-informative, accounting for 22.60% of the total (Tables S5 and S6). Maximum likelihood analysis under the GTR+ASC model recovered the 16 sampled *Blastus* accessions as a strongly supported monophyletic clade clearly separated from the four outgroups (UFBS = 100; Figure 9).

Within *Blastus*, the SNP tree recovered two major lineages, both of which received strong support. One lineage comprised *B. cochinchinensis*, *B. setulosus*, *B. tenuifolius*, and *B. mollissimus*, all of which possess axillary inflorescences. The second lineage comprised *B. borneensis* and all sampled members of the terminal inflorescence group. Within this lineage, *B. borneensis* was recovered as sister to the terminal inflorescence clade. The remaining sampled taxa with axillary inflorescences formed a strongly supported clade, but the traditional axillary inflorescence group as a whole was not monophyletic because *B. borneensis* was placed outside this clade.

The sampled members of the terminal inflorescence group formed a strongly supported monophyletic clade. Within this clade, the *B. pauciflorus* and *B. cavaleriei* groups were recovered as two strongly supported sister clades. Within the *B. pauciflorus* group, *B. pauciflorus* was sister to the clade comprising the remaining members, and *B. ernae* was sister to the clade comprising *B. longiflorus* and the two accessions of *B. longiflorus* var. *apricus*. The two accessions of *B. longiflorus* var. *apricus* did not form a monophyletic clade. The sampled members of the *B. cavaleriei* group also formed a strongly supported exclusive clade. Within this clade, the two accessions of *B. dunnianus* occupied separate positions and did not form an exclusive lineage. *Blastus dunnianus* 025 was sister to the clade comprising the remaining members of the group, whereas *B. dunnianus* 054 was sister to *B. cavaleriei* var. *tomentosus* 070, and the clade formed by these two accessions was in turn sister to *B. cavaleriei* 021.

Within the strongly supported clade comprising the remaining sampled taxa with axillary inflorescences, *B. cochinchinensis* was sister to the clade comprising *B. setulosus*, *B. tenuifolius*, and *B. mollissimus*. The two accessions of *B. setulosus* formed an exclusive clade that was sister to the lineage comprising *B. tenuifolius* and *B. mollissimus*. The two accessions of *B. mollissimus* also formed an exclusive clade, which was sister to *B. tenuifolius*.

Overall, the genomic SNP phylogeny showed the clearest correspondence with the morphological groupings among the molecular datasets analyzed, although this correspondence was incomplete. It supported the sampled members of the terminal inflorescence group as a monophyletic clade and recovered the *B. pauciflorus* and *B. cavaleriei* groups as two strongly supported sister lineages. However, the traditional axillary inflorescence group was not monophyletic because *B. borneensis* did not group with the remaining axillary inflorescence taxa. In addition, the sampled accessions of *B. longiflorus* var. *apricus* and *B. dunnianus* did not form exclusive lineages, indicating incomplete correspondence between the SNP topology and the current morphological delimitations at the species and varietal levels.

### 3.3. Correspondence Between Morphological Characters and Molecular Lineages

Comparison across the different molecular datasets revealed partial correspondence between the principal morphological groupings and the recovered molecular lineages. The sampled members of the terminal inflorescence group were recovered as a monophyletic lineage in both the plastome and genomic SNP trees. By contrast, the traditional axillary inflorescence group was not recovered as monophyletic in either the plastome or genomic SNP tree. In the SNP tree, *B. borneensis* was resolved as sister to the terminal inflorescence clade rather than grouping with the remaining sampled taxa possessing axillary inflorescences.

Within the terminal inflorescence group, the *B. cavaleriei* group formed an exclusive lineage in both the nrDNA and SNP trees, whereas the *B. pauciflorus* group formed an exclusive lineage in the plastome and SNP trees. In the SNP tree, these two informal groups were recovered as two strongly supported sister lineages. The two groups differed most consistently in the distribution of yellow glands on the abaxial leaf surface and in the shape and degree of reflexion of the calyx lobes. This combination of characters corresponded closely to the two principal lineages recovered in the SNP tree. By contrast, indumentum type and development varied considerably within each group and corresponded less consistently to the principal molecular lineages.

## 4. Discussion

### 4.1. Phylogenetic Position and Monophyly of Blastus

Previous phylogenetic studies of Melastomataceae and Sonerileae often included *Blastus* as an allied genus but generally sampled only one or a few species. Consequently, these studies primarily addressed the phylogenetic position of *Blastus* rather than comprehensively testing its monophyly and intrageneric relationships. Clausing et al. [41] included *B. borneensis* in a phylogenetic analysis of Melastomataceae based on plastid markers and recovered *Blastus* as closely related to *Phyllagathis*. Subsequent studies with broader sampling of Melastomataceae or Sonerileae similarly indicated that *Blastus* is closely related to genera such as *Phyllagathis*, *Fordiophyton*, and *Bredia* [42]. Based on combined analyses of ITS, *ndhF*, and *rpl16*, Zeng et al. [43] also recovered *Blastus* in close association with these genera. Zhou et al. [44], who included five species of *Blastus* in a phylogenetic study of *Bredia* and *Phyllagathis*, recovered *Blastus* as closely related to *Plagiopetalum* and *Phyllagathis*. A close relationship with *Bredia* and *Phyllagathis* was also reported when *B. cochinchinensis* was included as an allied taxon in a study describing a new species of *Bredia* [45]. Collectively, these studies consistently placed *Blastus* among a group of closely related genera in Sonerileae, but its monophyly remained insufficiently tested because of limited sampling within the genus.

The present study substantially expanded sampling within *Blastus* and evaluated its phylogenetic position using a broadly sampled ITS dataset, an expanded nrDNA cistron dataset, plastome data, and genomic SNP data. Both the ITS and nrDNA analyses recovered the sampled *Blastus* accessions as a strongly supported monophyletic clade. In the ITS phylogeny, *Blastus* was sister to a clade comprising *Phyllagathis cavaleriei* and *Fordiophyton faberi* (UFBS = 92, PP = 1.00). The same relationship was recovered with strong support in the expanded nrDNA tree (UFBS = 93, PP = 1.00). The genomic SNP analysis, which included 16 representative *Blastus* accessions and four related taxa used as outgroups, also strongly supported the monophyly of the sampled *Blastus* accessions. Thus, the ITS and expanded nrDNA analyses provided consistent nuclear ribosomal support, whereas the genomic SNP analysis supplied additional evidence from broader sampling of the nuclear genome. Together, these results provide substantially stronger support for the monophyly of the sampled *Blastus* accessions under the present taxonomic sampling than was available from previous studies.

By contrast, the plastome phylogeny did not recover *Blastus* as monophyletic, with *Fordiophyton faberi* and *Phyllagathis cavaleriei* placed between two lineages of the sampled *Blastus* accessions. This result indicates marked incongruence between the plastid and nuclear phylogenetic signals. Given the consistent support from the nuclear ribosomal and genomic SNP analyses, the plastome topology is more appropriately interpreted as evidence of cytonuclear incongruence than as definitive evidence against the monophyly of *Blastus*. Overall, the available nuclear evidence supports *Blastus* as a monophyletic lineage under the present sampling of Sonerileae, whereas the evolutionary processes underlying the conflicting plastome topology require further investigation.

### 4.2. Cytonuclear Incongruence and Differences in Phylogenetic Resolution Among Datasets

The broadly sampled ITS analysis, expanded nrDNA cistron analysis, and genomic SNP analysis consistently supported the monophyly of the sampled *Blastus* accessions, but differed substantially in their ability to resolve relationships within the genus. ITS has been widely used in plant phylogenetic studies. The limited number of informative characters available from this region may be insufficient to resolve relationships among closely related taxa or lineages that diverged over a short period. Previous studies of Asian Sonerileae similarly showed that nrITS provided limited resolution for several complex phylogenetic relationships [44,46]. In the present study, the ITS analysis strongly supported the monophyly of the sampled *Blastus* accessions but provided limited resolution for many relationships among closely related taxa and did not consistently recover the principal morphological groups. The expanded nrDNA dataset, comprising 18S, ITS1, 5.8S, ITS2, and 28S, increased nuclear ribosomal sequence coverage and recovered the sampled members of the *B. cavaleriei* group as an exclusive lineage. Nevertheless, several internal relationships remained weakly supported or unresolved, indicating that nuclear ribosomal data alone were insufficient to resolve all recent divergences within *Blastus*.

Compared with the two nuclear ribosomal datasets, the genomic SNP dataset substantially improved the resolution of relationships within *Blastus*. Genomic SNP data sample variation across numerous regions of the nuclear genome and can therefore provide considerably more phylogenetic information for resolving recent divergences and relationships among closely related taxa [11,12,47]. In the present study, the SNP analysis strongly supported the monophyly of the sampled *Blastus* accessions and clearly resolved the principal nuclear genomic lineages within the genus. The *B. pauciflorus* and *B. cavaleriei* groups were recovered as two strongly supported sister lineages. However, the sampled accessions assigned to *B. longiflorus* var. *apricus* and *B. dunnianus* did not form an exclusive lineage. In addition, the traditional axillary inflorescence group was not recovered as monophyletic because *B. borneensis* was resolved as sister to the terminal inflorescence clade rather than grouping with the remaining sampled taxa possessing axillary inflorescences. These results indicate that broader sampling of the nuclear genome substantially improved the resolution of the principal lineages within *Blastus*, although some species and varietal boundaries defined primarily by morphology were not fully supported by the SNP topology.

By contrast, the plastome analysis did not recover the sampled *Blastus* accessions as monophyletic. The plastome tree divided them into two lineages, with *Fordiophyton faberi* and *Phyllagathis cavaleriei* placed between the two *Blastus* lineages. Plant plastomes are generally inherited from one parent and largely behave as a single linked genomic compartment. Consequently, plastome phylogenies primarily record the evolutionary history of the plastid lineage and, in groups with complex evolutionary histories, may not fully reflect nuclear genomic history or the evolutionary history of the species involved [46,48,49]. Several relationships that conflicted with the nuclear phylogenies received strong support in the plastome analysis. The observed incongruence is therefore unlikely to be explained solely by limited phylogenetic information or stochastic error. Instead, it suggests that the plastid and principal nuclear genomic lineages may have recorded different evolutionary histories.

Similar discordance between nuclear and plastid datasets has been reported in Asian Sonerileae, where phylogenetic conflict has been associated with missing data bias, stochastic noise from loci containing limited phylogenetic information, incomplete lineage sorting, and historical hybridization or introgression [46]. More generally, plastid capture has also been proposed as a potential cause of cytonuclear incongruence in plants [50,51,52,53]. Incomplete lineage sorting can result in ancestral polymorphisms being retained and sorted differently among descendant lineages. Historical hybridization, introgression, or plastid capture can instead introduce a plastid lineage into a taxon with a different nuclear genomic background. These processes may explain why the plastome tree differs markedly from the nuclear phylogenies in its reconstruction of the monophyly of the sampled Blastus accessions and several principal relationships within the genus. However, the present study did not directly test these processes, and the available evidence is insufficient to determine their relative contributions. The observed cytonuclear incongruence therefore suggests that *Blastus* and its allied taxa may have experienced a complex evolutionary history, but it does not constitute direct evidence for any particular process, including incomplete lineage sorting, hybridization, introgression, or plastid capture. Future studies should expand sampling at the population and geographic levels and incorporate multispecies coalescent analyses together with explicit tests of gene flow and introgression to clarify the evolutionary mechanisms underlying this phylogenetic incongruence.

### 4.3. Morphological Differentiation and Its Phylogenetic Significance

Morphological characters in *Blastus* differ in their taxonomic value across taxonomic levels. Characters such as the shrubby habit, basal venation, tetramerous flowers, and general hypanthium morphology collectively define the broad morphological range of the genus. However, because these characters are shared by many taxa within *Blastus*, they provide limited information for resolving the principal lineages within the genus. By contrast, inflorescence position and architecture, the distribution of yellow glands on the abaxial leaf surface, calyx lobe morphology, anther length and basal morphology show greater differentiation within the genus. Their taxonomic value should therefore be evaluated separately at different taxonomic levels and in conjunction with molecular evidence.

Inflorescence position and basic architecture represent one of the most conspicuous patterns of morphological differentiation within *Blastus* and provided the principal basis for the two broad descriptive assemblages proposed by Diels [23]. The molecular results show that this division has partial, but not complete, phylogenetic support. The sampled members of the terminal inflorescence group formed a monophyletic lineage in both the plastome and genomic SNP trees and received strong support in the SNP analysis. With the exception of *B. borneensis*, the remaining sampled taxa possessing axillary inflorescences also formed a strongly supported clade in the SNP tree. This correspondence suggests that inflorescence position reflects an important pattern of morphological differentiation within *Blastus* and corresponds closely to the principal nuclear genomic lineages in most of the sampled taxa. It therefore has practical value for recognizing the major patterns of differentiation within the genus. However, *B. borneensis* did not group with the remaining sampled taxa possessing axillary inflorescences but was recovered as sister to the terminal inflorescence clade. This placement disrupts a strict correspondence between inflorescence position and the principal nuclear genomic lineages. Inflorescence position is therefore phylogenetically informative and diagnostically useful, but it does not uniquely define every principal lineage. It should be retained as a descriptive character rather than used alone to establish two formal and mutually exclusive infrageneric taxa.

Within the terminal inflorescence group, the *B. pauciflorus* group and the *B. cavaleriei* group were proposed before the molecular analyses as two informal working units based on distinct combinations of morphological characters. They were distinguished primarily by the distribution of yellow glands on the abaxial leaf surface and by the shape and degree of reflexion of the calyx lobes. The broadly sampled ITS and expanded nrDNA analyses provided different levels of nuclear ribosomal support for this morphological division. Because ITS contained relatively limited phylogenetic information, members of the two groups were intermingled in the ITS tree and did not form separate lineages. The expanded nrDNA analysis recovered the sampled members of the *B. cavaleriei* group as an exclusive lineage, whereas the sampled members of the *B. pauciflorus* group were still not recovered as monophyletic. The genomic SNP analysis, which incorporated a large number of nuclear genomic variants, recovered the two morphological groups as two strongly supported sister lineages. Thus, among the three nuclear datasets examined here, the correspondence between the molecular lineages and morphology-based groups became increasingly clear as nuclear genomic coverage and the amount of independent phylogenetic information increased. The plastome analysis recovered the sampled members of the *B. pauciflorus* group as an exclusive lineage but did not recover those of the *B. cavaleriei* group as an exclusive lineage. Thus, the two groups were not supported consistently across different genomic compartments. Comparable studies in Asian Sonerileae have similarly shown that morphological circumscriptions may correspond only partly with nrITS and plastid phylogenies and that broader nuclear evidence may be needed before formal taxonomic changes are proposed [44,53]. The available evidence therefore supports treating the *B. pauciflorus* and *B. cavaleriei* groups as informal working units with clear morphological foundations and correspondence with the genomic SNP phylogeny. Whether they warrant formal infrageneric rank requires broader taxonomic and population sampling together with congruent evidence from multiple genomic compartments.

At the species and varietal levels, differences in character stability were more pronounced. The auriculate leaf bases and sessile or subsessile leaves clearly distinguish *B. auriculatus*, whereas the markedly shorter anthers of *B. pauciflorus* also have high diagnostic value [23]. The shape and degree of reflexion of the calyx lobes are informative for distinguishing some closely related taxa, although intermediate forms occur in some specimens and reduce the reliability of these characters when used alone. By contrast, the density and development of spreading glandular hairs, glandular setae, and other forms of indumentum vary considerably among individuals and populations and do not consistently correspond to the lineages recovered for some species and varieties. Indumentum characters are therefore more appropriately treated as supplementary evidence and should not be used alone as decisive criteria for delimiting closely related species or varieties.

### 4.4. Taxonomic Implications and Recommendations

The combined nuclear evidence supports retaining the current generic circumscription of *Blastus* under the present sampling. The broadly sampled ITS and expanded nrDNA analyses provided consistent nuclear ribosomal support for the monophyly of the sampled *Blastus* accessions, while the genomic SNP analysis provided additional support based on broader sampling across the nuclear genome.

At the infrageneric level, a formal classification should not be established solely on the traditional distinction between axillary and terminal inflorescences, because the axillary inflorescence group was not recovered as monophyletic. Inflorescence position remains diagnostically useful and corresponds closely to some of the principal nuclear genomic lineages, but it does not delimit all major evolutionary lineages within the genus. Within the terminal inflorescence group, the *B. pauciflorus* group and *B. cavaleriei* group are distinguished by distinct combinations of morphological characters, and their sampled members were recovered as strongly supported sister lineages in the genomic SNP tree. However, support for the two groups was not consistent across the broadly sampled ITS, expanded nrDNA, and plastome trees. We therefore recommend retaining them as informal working units for species identification, morphological comparison, and future taxonomic revision without assigning them formal infrageneric rank at present.

The present results also indicate that the broad circumscription of *B. pauciflorus* sensu lato does not adequately represent the correspondence between morphological differentiation and nuclear genomic lineages among the relevant sampled taxa. In the SNP tree, these taxa formed two strongly supported sister lineages corresponding broadly to the *B. pauciflorus* and *B. cavaleriei* groups rather than a single lineage with little internal differentiation. The circumscription of *B. pauciflorus* sensu lato therefore requires further reassessment.

At the species and varietal levels, the sampled accessions of *B. longiflorus* var. *apricus* and *B. dunnianus* did not form exclusive lineages, indicating that their current delimitations are not fully supported by the genomic SNP data. The accessions with different phylogenetic placements were collected from different localities, raising the possibility that some of the observed genetic variation is geographically structured. However, limited sampling at the population level does not permit discrimination between geographic differentiation and uncertainty in the current taxonomic boundaries. In particular, species or varietal boundaries based primarily on differences in indumentum density or the development of glandular hairs and glandular setae should be treated with caution. The available evidence is insufficient either to confirm these boundaries or to justify synonymization of the corresponding names. These names should therefore be retained provisionally, and the relevant taxa should be prioritized for broader population sampling and further taxonomic reassessment.

Accordingly, we recommend retaining the current generic circumscription of *Blastus*, treating the axillary and terminal inflorescence groups only as descriptive morphological categories, and retaining the *B. pauciflorus* and *B. cavaleriei* groups as informal working units. Because sampling at the population level remains limited, no formal synonymizations, changes in rank, or new combinations are proposed here.

## 5. Conclusions

The current generic circumscription of *Blastus* is supported by the present sampling. However, inflorescence position alone does not support the establishment of two formal infrageneric taxa because the traditional axillary inflorescence group is not monophyletic. The axillary inflorescence group and the terminal inflorescence group should therefore be treated only as descriptive morphological categories. Within the terminal inflorescence group, the *B. pauciflorus* and *B. cavaleriei* groups can be retained as informal working units because they correspond broadly to two strongly supported nuclear genomic lineages and are distinguished by the distribution of yellow glands on the abaxial leaf surface and by calyx lobe morphology. The broad circumscription of *B. pauciflorus* adopted in the *Flora of China* requires reassessment because it does not adequately represent the principal morphological and nuclear genomic differentiation among the relevant taxa. However, the boundaries of *B. longiflorus* var. *apricus* and *B. dunnianus* remain insufficiently resolved to justify formal nomenclatural changes. Broader sampling at the population and geographic levels is required before synonymization, changes in rank, or new combinations are proposed.

## Data Availability

The annotated plastomes, ITS, and nrDNA sequences of the 28 taxa studied have been submitted to NGDC (https://ngdc.cncb.ac.cn/genbase (accessed on 16 June 2026)) with accession numbers that can be found in Table S1.

## Supplementary Materials

The following supporting information can be downloaded at: https://www.mdpi.com/article/10.3390/plants15162526/s1, Figure S1: Morphological characteristics of *Blastus cochinchinensis*; Figure S2: Morphological characteristics of *Blastus tenuifolius*; Figure S3: Morphological characteristics of *Blastus setulosus;* Figure S4: Morphological characteristics of *Blastus auriculatus;* Figure S5: Morphological characteristics of *Blastus borneensis*; Figure S6: Morphological characteristics of *Blastus mollissimus*; Figure S7: Morphological characteristics of *Blastus pauciflorus;* Figure S8: Floral morphology of *Blastus longiflorus*; Figure S9: Inflorescence and floral morphology of *Blastus longiflorus* var. *apricus*; Figure S10: Inflorescence and floral morphology of *Blastus ernae*; Figure S11: Morphological characteristics of *Blastus cavaleriei*; Figure S12: Inflorescence and floral morphology of *Blastus cavaleriei* var. *Tomentosus;* Figure S13. Floral morphology of *Blastus dunnianus*; Figure S14: Morphological characteristics of *Blastus dunnianus* var. *glandulosetosus*; Figure S15: Phylogenetic relationships of *Blastus* and allied taxa based on the complete nuclear ITS dataset. Table S1: Material sample source and information; Table S2: Comparison of morphological characteristics among sampled taxa of *Blastus*; Table S3: Morphological comparison of taxa in the *B. pauciflorus* group; Table S4: Morphological comparison of taxa in the *B. cavaleriei* group; Table S5: Summary statistics of molecular datasets used for phylogenetic analyses in this study; Table S6: Missing and ambiguous base statistics for each sample in the final SNP matrix.
