# Supplementary material for: Morphological and Molecular Evidence for Infrageneric Relationships in Blastus (Melastomataceae)
Source: Plants (Basel). 2026 Aug 20;15(16):2526. doi: 10.3390/plants15162526 (PMC13516078; doi:10.3390/plants15162526)
Supplement: Supplementary file 1 [file plants-15-02526-s001.zip › Figure supplementary.pdf]

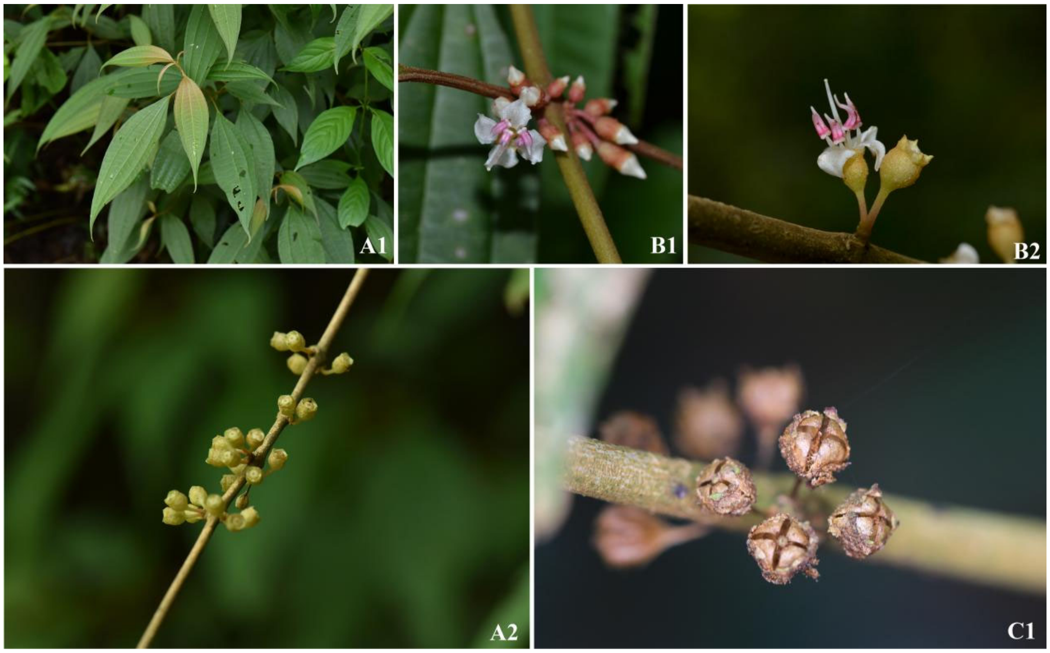

Figure S1. Morphological characteristics of *Blastus cochinchinensis.*
(A1, A2) Population from Damingshan, Guangxi, China: A1. leaf; A2. fruit. (**B1, B2**) Population from Lianhuashan, Huidong, Guangdong, China, showing floral morphology. (**C1**) Population from Guiping, Guangxi, China, showing fruit.

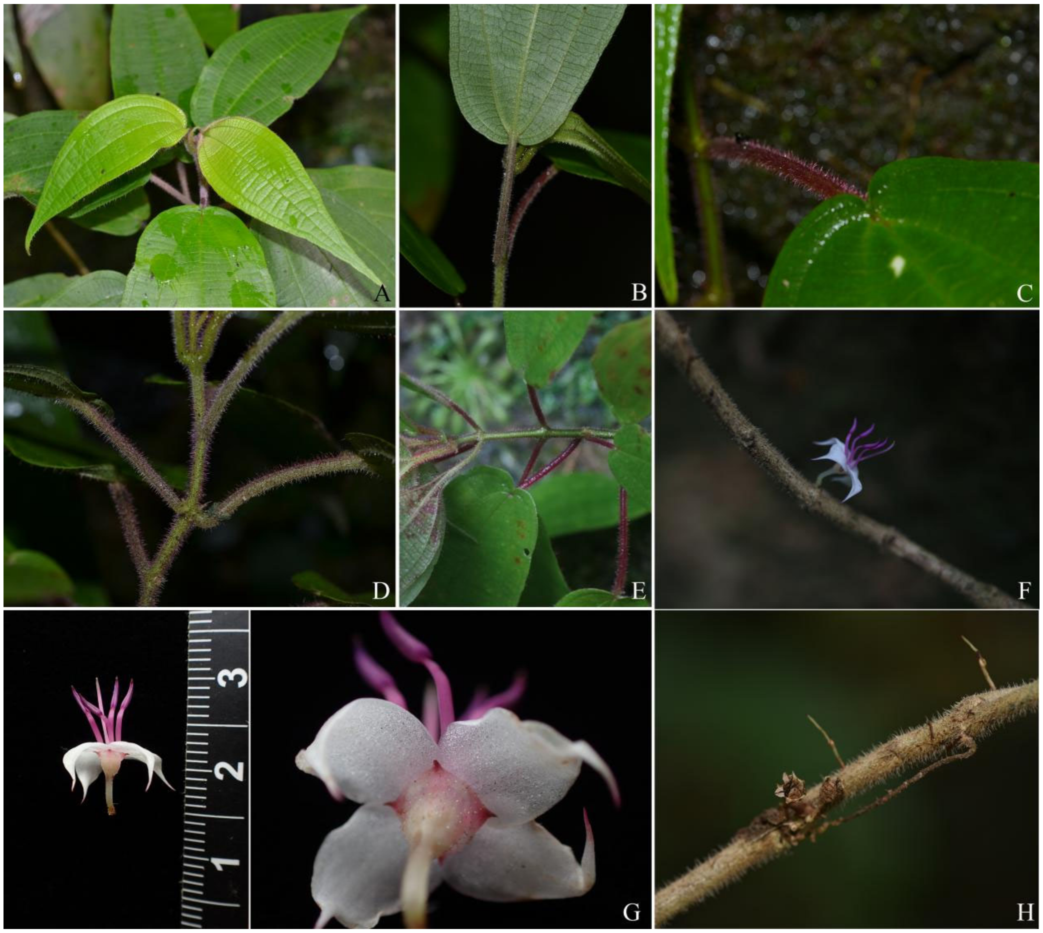

Figure S2. Morphological characteristics of *Blastus tenuifolius.*

(**A, B**) Leaves; (**C–E**) branches and indumentum; (**F, G**) flowers; (**H**) fruit. Material from Ehuangzhang Nature Reserve, Yangchun, Guangdon, Chinag.

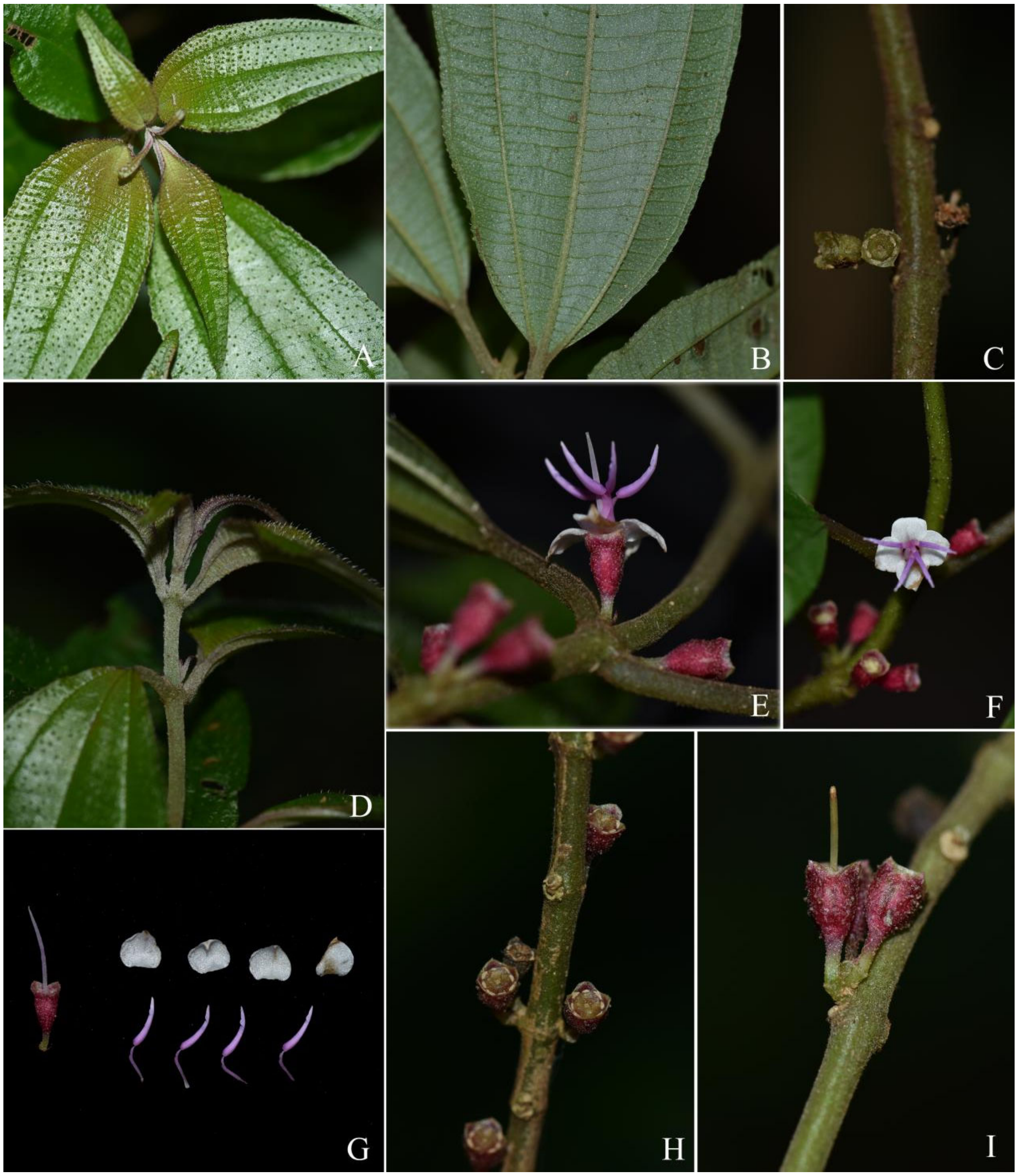

Figure S3. Morphological characteristics of *Blastus setulosus.*

(**A, B, D**) Leaves; (**C, H, I**) fruits; (E–G) floral morphology. Material from Huatan Forest Farm, Yangchun, Guangdong, China.

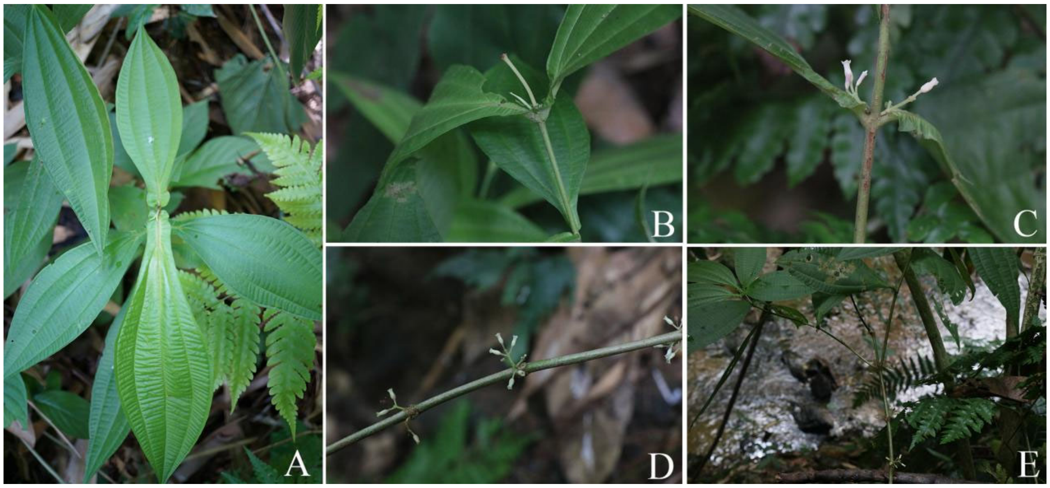

Figure S4. Morphological characteristics of *Blastus auriculatus.*
(**A, B**) Leaves; (**C, D**) inflorescences; (**E**) branch. Material from Hekou, Yunnan, China.

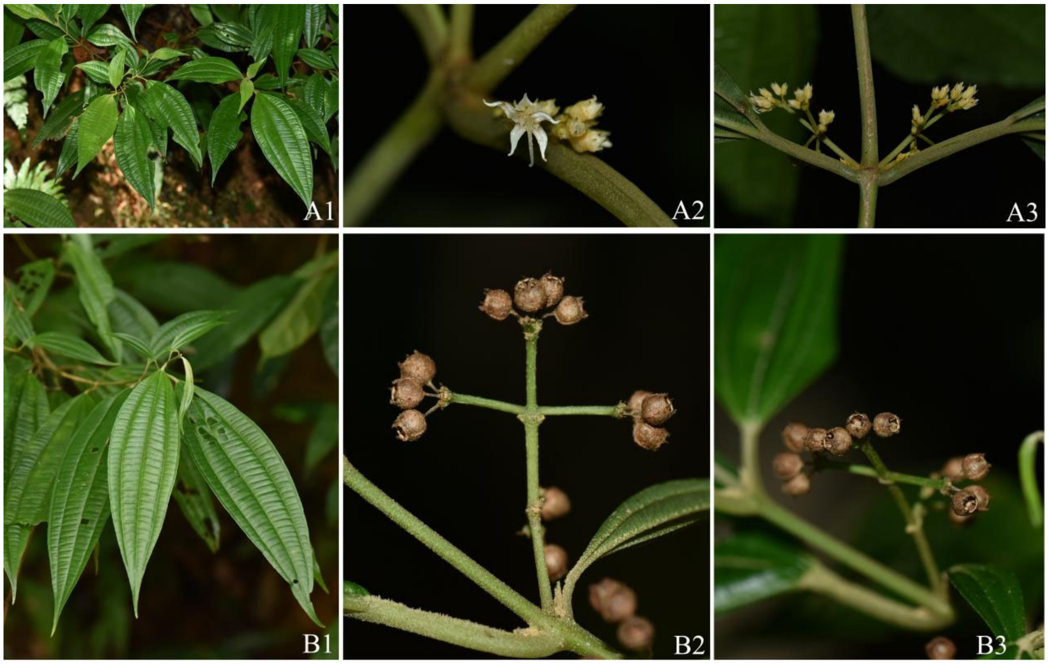

Figure S5. Morphological characteristics of *Blastus borneensis.*
(**A1, B1**) Leaves; (**A2, A3**) inflorescences and flowers; (**B2, B3**) fruits. (**A1–A3**) Jianfengling, Ledong, Hainan, China; (**B1–B3**) Kubah National Park, Sarawak, Malaysia.

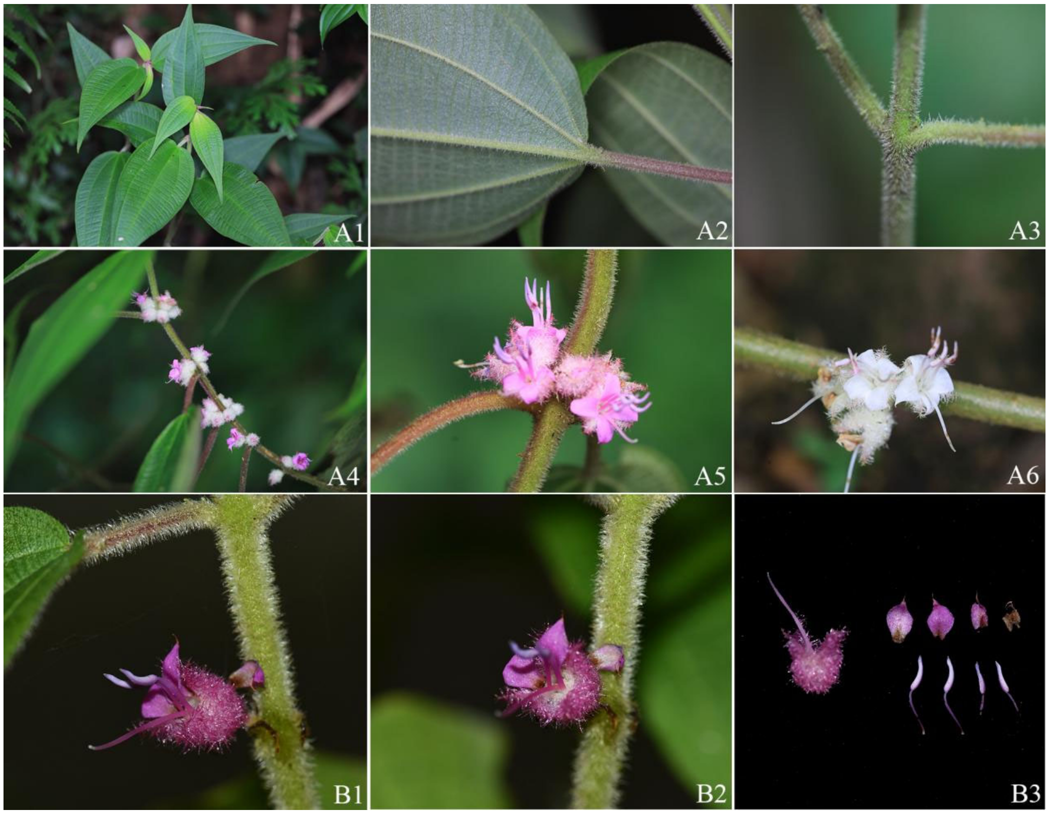

Figure S6. Morphological characteristics of *Blastus mollissimus.*

(**A1, A2**) Leaves; (**A3**) branch and indumentum; (**A4–B3**) floral morphology. (**A1–A6**) form Liuxiang Township, Jinxiu, Guangxi, China; (**B1–B3**) from Shibahe, Zijing Town, Guiping, Guangxi, China.

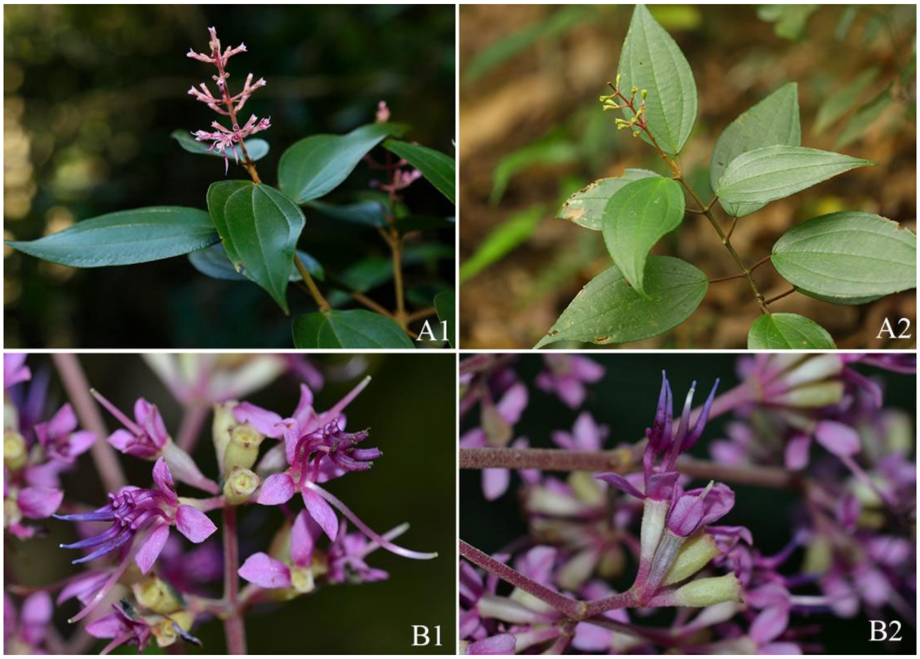

Figure S7. Morphological characteristics of *Blastus pauciflorus.*
(A1–A2) Population from Hong Kong, China, showing habit, leaves, and inflorescences; (**B1–B2**) population from Huidong, Guangdong, China, showing floral morphology. A1 and A2 were provided by the Hong Kong Herbarium.

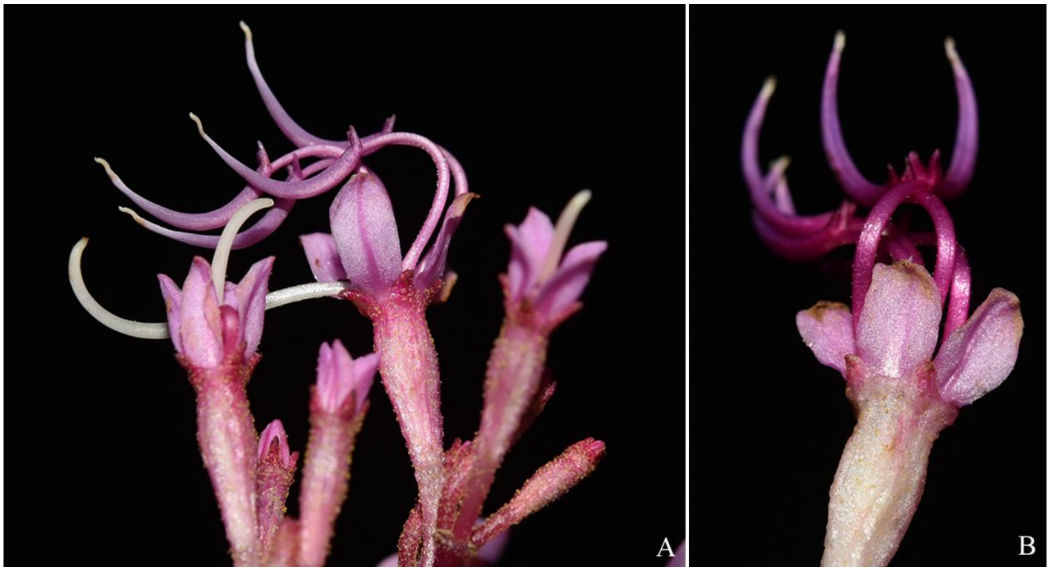

Figure S8. Floral morphology of *Blastus longiflorus.*
(A, B) Flowers and calyx lobes. Material from Tianjingshan, Shaoguan, Guangdong, China.

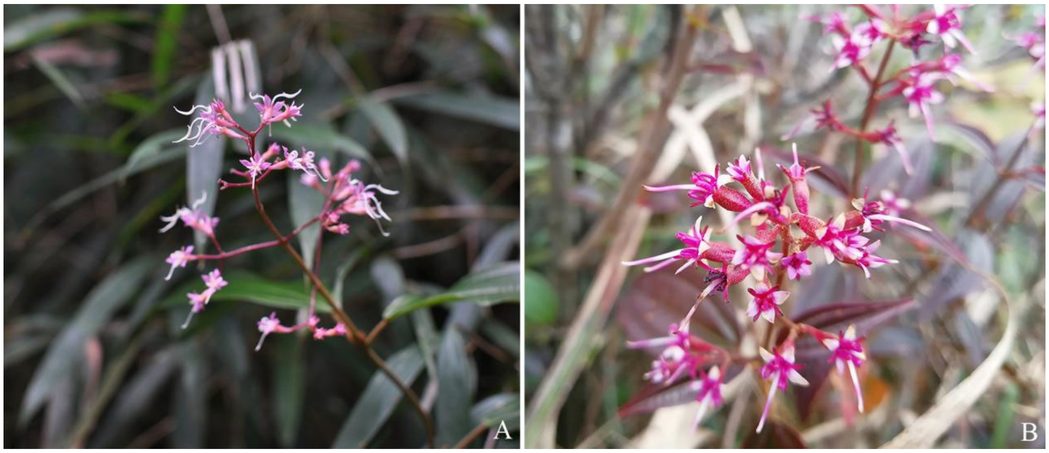

Figure S9. Inflorescence and floral morphology of *Blastus longiflorus* var. *apricus.*
(**A**) Population from Nankunshan, Huizhou, Guangdong, China; (**B**) population from Yequgou, Heyuan, Guangdong, China.

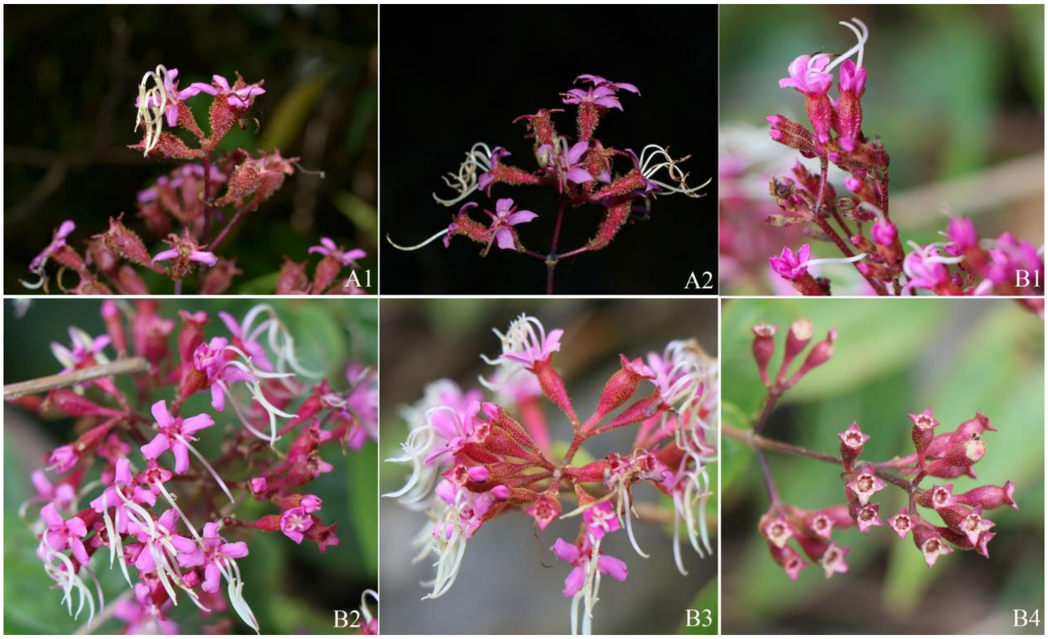

Figure S10. Inflorescence and floral morphology of *Blastus ernae*.
(**A1–A2**) Population from Nankunshan, Huizhou, Guangdong, China; (**B1–B4**) population from Tanwu, Zhongzhou Town, Huaiji, Guangdong, China.

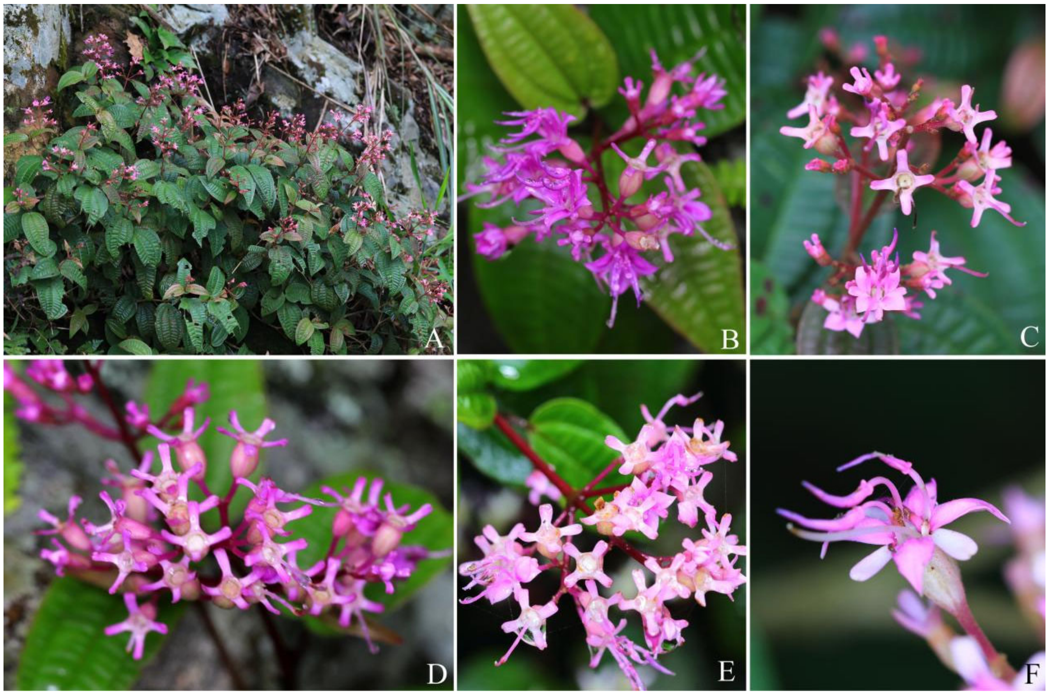

Figure S11. Morphological characteristics of *Blastus cavaleriei.*
(A–F) inflorescences, flowers, and calyx lobes. Material from Damingshan, Guangxi, China.

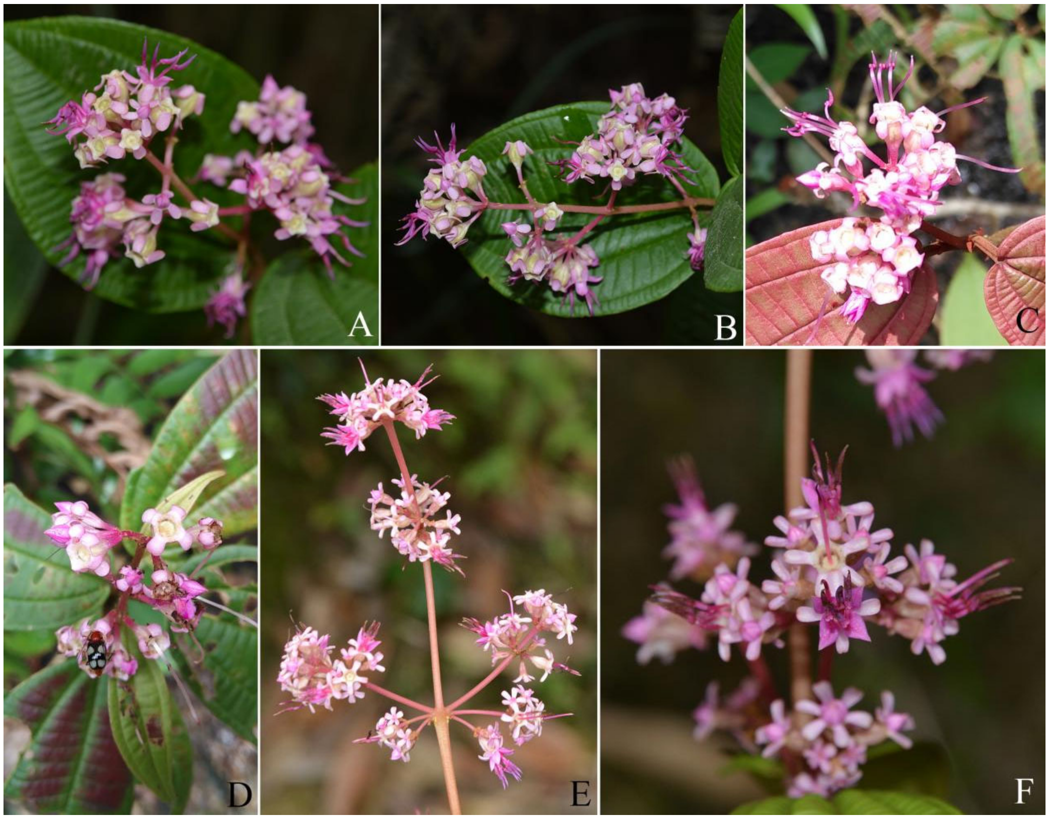

Figure S12. Inflorescence and floral morphology of *Blastus cavaleriei* var. *tomentosus.*
(**A–H**) Inflorescences, flowers, and calyx lobes. Material from Qixingding, Fengkai, Zhaoqing, Guangdong, China.

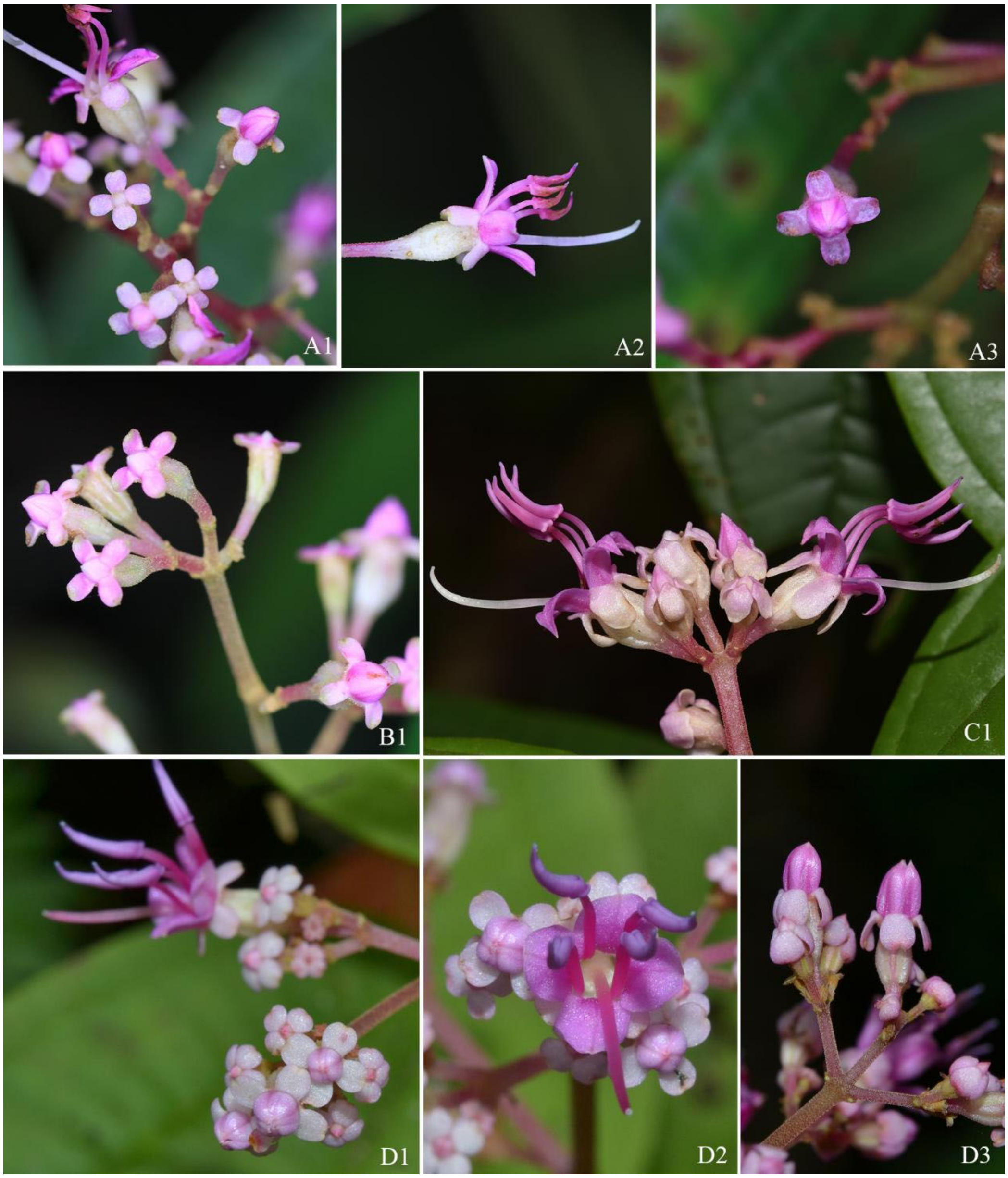

Figure S13. Floral morphology of *Blastus dunnianus*.
(**A1–A3**) Population from Shengtangshan, Jinxiu, Guangxi, China; (**B1**) population from Yinshan Park, Jinxiu, Guangxi, China; (**C1**) population from Jinggangshan, Jiangxi, China; (**D1–D3**) population from Nankunshan, Huizhou, Guangdong, China.

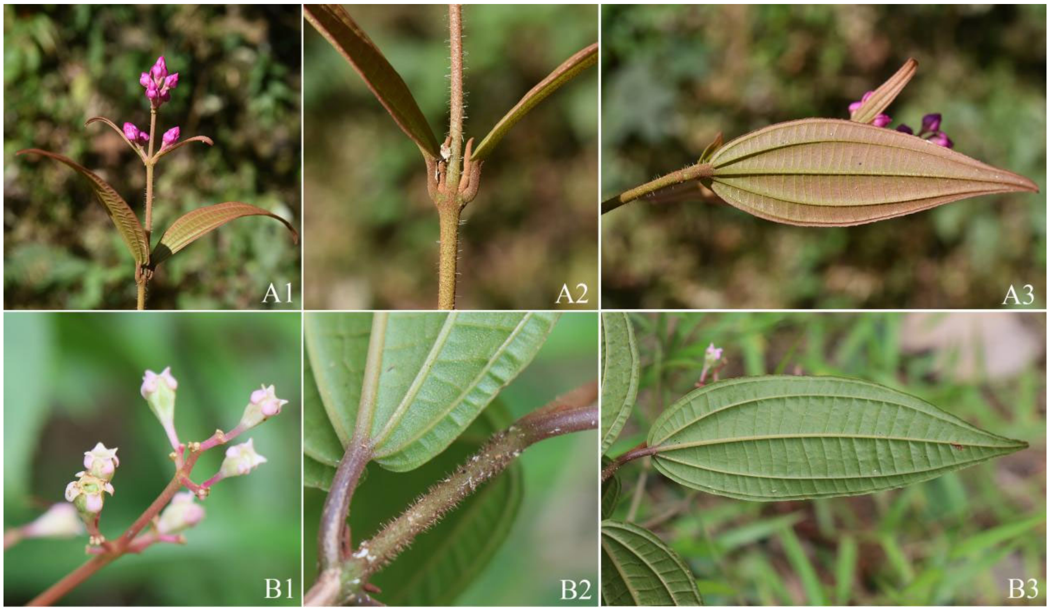

Figure S14. Morphological characteristics of *Blastus dunnianus* var. *glandulosetosus*. (**A1–A3**) Population from Mangshan, Hunan, China, showing inflorescences, abaxial leaf surface, and indumentum; (**B1–B3**) population from Tanwu, Zhongzhou Town, Huaiji, Guangdong, China, showing inflorescences, abaxial leaf surface, and indumentum.

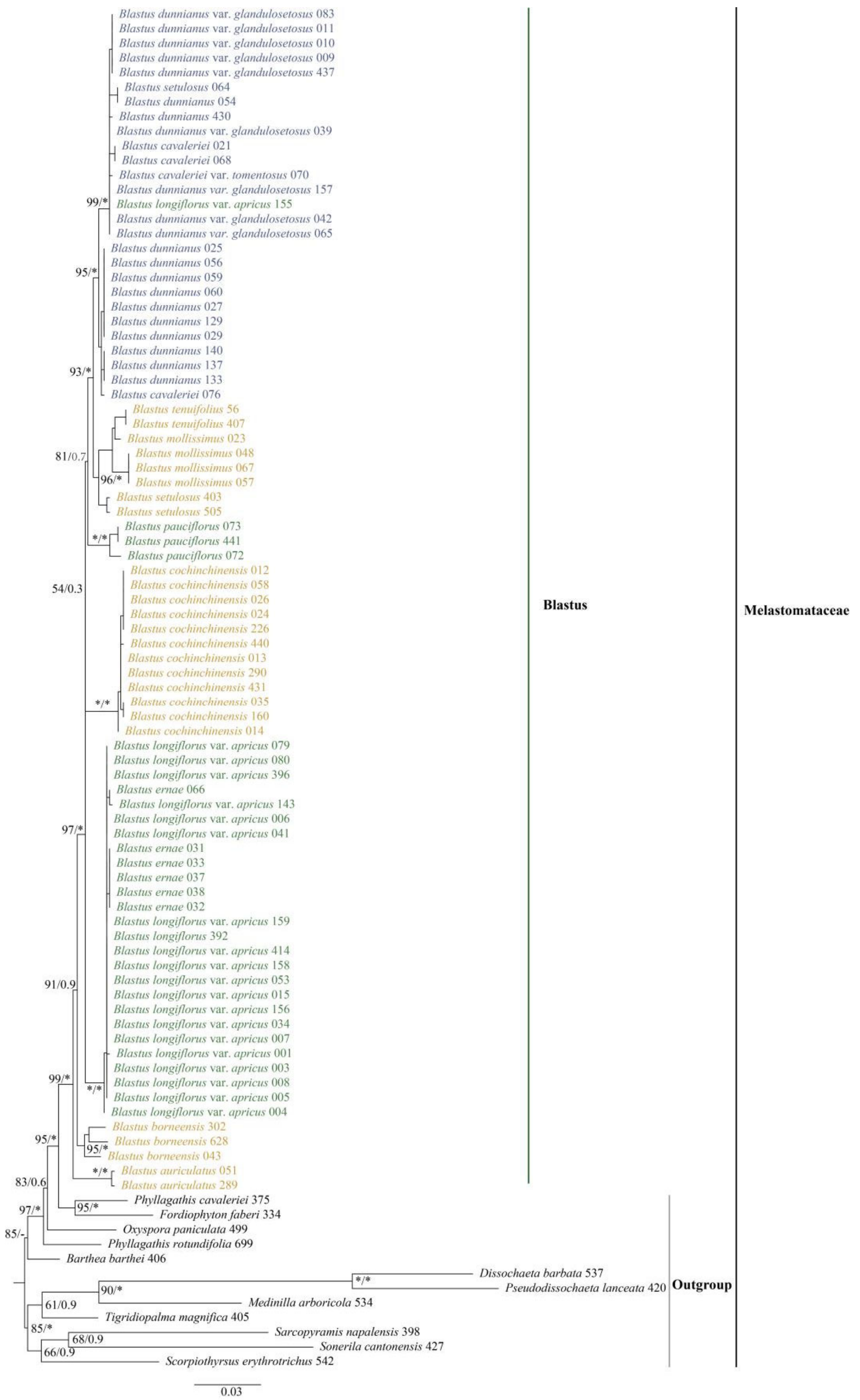

Figure S15. Phylogenetic relationships of *Blastus* and allied taxa based on the complete nuclear ITS dataset. Numbers near the branches indicate ultrafast bootstrap support and Bayesian posterior probabilities (UFBS/PP), respectively. A dash (–) indicates topological incongruence between the ML and BI trees at that node. Colors indicate the morphological assignments of the sampled taxa: yellow, axillary-inflorescence group; green, the B. pauciflorus group; and blue, the B. cavaleriei group. The scale bar represents the expected

number of substitutions per site. Asterisks indicate maximum support (UFBS = 100 or PP = 1.00), and dashes indicate that the corresponding node was not recovered in the Bayesian inference tree.
